# Cysteine cathepsins: their role in tumor progression and recent trends in the development of imaging probes

**DOI:** 10.3389/fchem.2015.00037

**Published:** 2015-06-23

**Authors:** Reik Löser, Jens Pietzsch

**Affiliations:** ^1^Department of Radiopharmaceutical and Chemical Biology, Institute of Radiopharmaceutical Cancer Research, Helmholtz-Zentrum Dresden-RossendorfDresden, Germany; ^2^Department of Chemistry and Food Chemistry, Technische Universität DresdenDresden, Germany

**Keywords:** cancer, carcinogenesis, extracellular enzymes, fluorescence-based probes, lysosomal cysteine proteases, metastasis, molecular imaging, radiotracers

## Abstract

Papain-like cysteine proteases bear an enormous potential as drug discovery targets for both infectious and systemic human diseases. The considerable progress in this field over the last two decades has also raised interest in the visualization of these enzymes in their native context, especially with regard to tumor imaging. After a short introduction to structure and general functions of human cysteine cathepsins, we highlight their importance for drug discovery and development and provide a critical update on the current state of knowledge toward their involvement in tumor progression, with a special emphasis on their role in therapy response. In accordance with a radiopharmaceutical point of view, the main focus of this review article will be the discussion of recently developed fluorescence and radiotracer-based imaging agents together with related molecular probes.

## Introduction: structural and biochemical considerations

**Figure 1:**
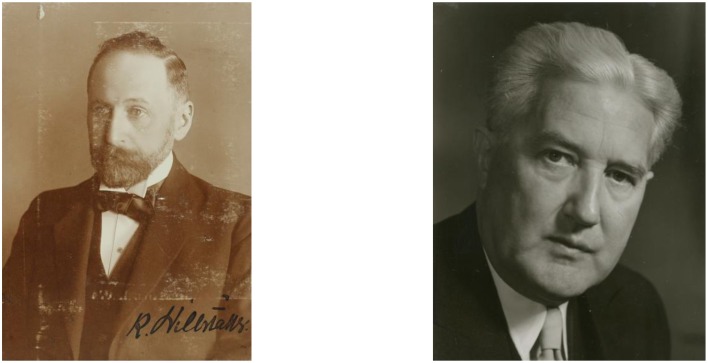
**Richard Willstätter (left) and Eugen Bamann (right), the coiners of term cathepsin**. © Archive of Deutsche Akademie der Naturforscher Leopoldina, M1 3416 and M1 4898.

**Figure 2:**
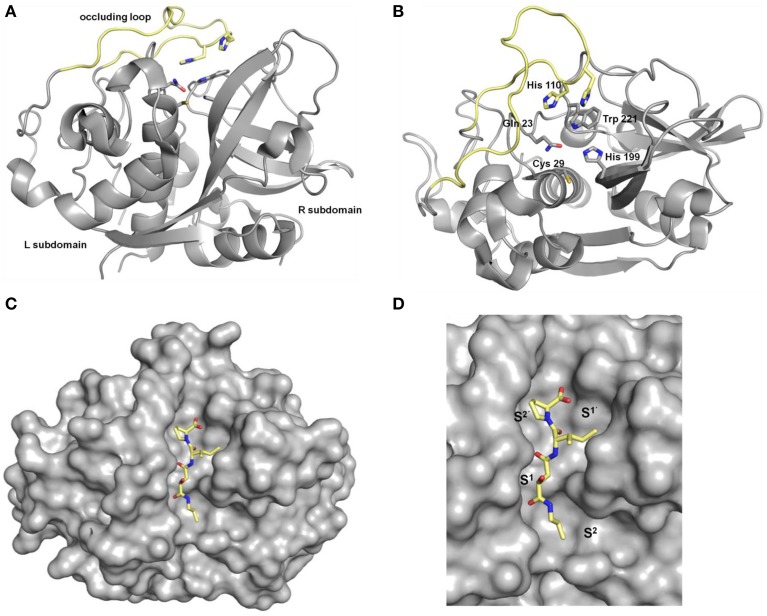
**Crystal structures of human cathepsin B. (A)** View from “front,” **(B)** view from “top”; the occluding loop is highlighted in yellow, residues Cys29, His199, Gln 23, and Trp221 correspond to Cys25, His159, Gln19 and Trp177 in papain, respectively, **(C**,**D**) cathepsin B (shown in gray surface) in complex with CA074 (**9a**, shown in sticks). Pictures were prepared with PyMOL (DeLano, W. L. *The PyMOL Molecular Graphics System*. Version 1.5.0.3 Schrödinger, LLC) using the PDB file 1QDQ (2.2 Å) (Yamamoto et al., 2000).

**Figure 3:**
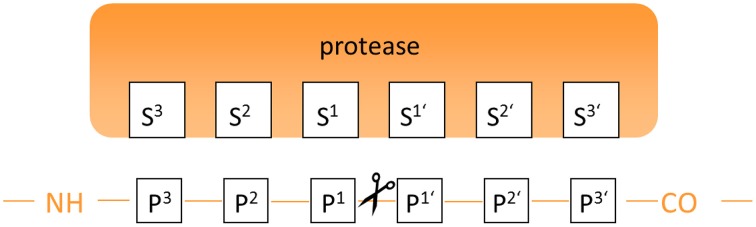
**Annotation of binding sites and amino acid residues for the description of the protease-substrate interaction**.

The term cathepsins was introduced by the famous chemist Richard Willstätter (1872–1942) and his PhD student Eugen Bamann (1900–1981) for the entirety of the intracellular proteases referring to their protein-degrading activity (greek καθεψειν = to digest, to boil) in the first half of the last century (Figure 1) (Willstätter and Bamann, 1929). In this sense, their physiological functions were for long time considered to be restricted to cellular protein catabolism. The class of proteases referred to as cathepsins represents a structurally heterogeneous group of enzymes. However, in humans the majority of them belong to the mechanistic class of the cysteine proteases as they contain a highly conserved cysteine residue in their active sites. Because they share a high degree of homology to the plant enzyme papain, the cysteine cathepsins are included in the C1 family of clan CA according to the MEROPS protease classification system (Rawlings et al., 2014)^1^. In addition to the 11 papain-like cathepsins B, C, F, H, K, L, O, S, V, W, and X, four non-cysteine cathepsins are present in humans, i.e., the serine proteases cathepsins A (also referred to as serine carboxypeptidase A) and G and the aspartic proteases cathepsins D and E (Kirschke, 2008). With the exception of cathepsin C, which is present as a homotetramer, all cysteine cathepsins are monomeric single domain enzymes with molar masses ranging between 24 and 28 kDa for the mature enzymes. As characteristic for all papain-like enzymes, their structure is composed of two subdomains referred to as the L (left)- and the R (right)-domains. The N-terminal L-domain is dominated by α-helical structures among which a long N-terminal α-helix that harbors the active-site cysteine residue at its beginning is the most striking element. The R-domain is located toward the C-terminus and is characterized by a β-barrel motif. The V-shaped active-site cleft is situated between the two subdomains with the residues cysteine 25, histidine 159, asparagine 175, and glutamine 19 (papain numbering) constituting the catalytic center. The binding sites that recognize the amino acid side chains on the peptidic substrates are located in alternating sequence on the L- and R-subdomains which requires the substrate to be present in the extended conformation for productive binding (McGrath, 1999; Brömme, 2001; Grzonka et al., 2001; Turk et al., 2001b, 2003, 2012; Lecaille et al., 2002; Stoka et al., 2005). Figure 2 explains the structure of papain-like cysteine proteases exemplarily for human cathepsin B. Within the S^2^ to S^1′^ binding sites (see Figure 3 for explanation) the contacts to the substrate comprise non-covalent interactions to the side-chain entities and hydrogen bonds to the peptidic backbone. The enzyme-substrate contacts beyond these sites, i.e., S^3^ and S^2′^ in the N- and C-terminal direction, respectively, are devoid of hydrogen bonds and less well-defined (Turk et al., 1997; Turk and Gunčar, 2003). Regarding the mechanism of catalysis, instructive insights have been gained from investigations on papain, which in principle can be translated to the other members of the clan CA proteases including the cysteine cathepsins. A catalytic triad is formed by cysteine 25, histidine 159, and asparagine 175. Due to the formation of a thiolate-imidazolium ion pair, the active-site cysteine is present as a permanent negatively charged nucleophile. The ion pair is stabilized by the asparagine 175 whose side chain carbonyl oxygen acts as an H-bond acceptor toward the imidazole NH of histidine 159, which results in an increased basicity of this imidazole moiety (Vernet et al., 1995). Further, stabilization of the thiolate arises by the fact that the active-site cysteine is located at the beginning of the N-terminal α-helix mentioned before, which enables the negative charge to be stabilized by the helix macrodipole (Rullmann et al., 1989). In addition, the thiolate-imidazolium ion pair is shielded from solvent by the side chain of tryptophan 177 (Gul et al., 2008). The stabilized negative charge renders the active-site cysteine capable of nucleophilic attack toward the peptide bond in the substrate resulting in the formation of a transient thiol ester. During this step, a tetrahedral intermediate is passed, whose oxygen-centered negative charge is stabilized by H-bond contacts to one of the side chain amide proton of glutamine 19 and the backbone NH of cysteine 25, the so called oxyanion hole. Upon collapse of this tetrahedral intermediate, a proton is transferred from the imidazolium moiety of histidine 159 to the nitrogen of the cleaved peptide bond which results in the release of the C-terminal cleavage product from the enzyme. In the following step, the neutral imidazole ring of the histidine acts as general base upon nucleophilic attack of the thiol ester by water under the formation of a second tetrahedral intermediate. The collapse of this intermediate releases the N-terminal cleavage product and restores the thiolate-imidazolium ion pair ready for another cycle of catalysis.

The majority of the human cysteine cathepsins act as endopeptidases. Exopeptidase activity is exhibited by the cathepsins B, C, H, and X. Several structural characteristics determine their action as endopeptidases (Turk and Gunčar, 2003; Novinec and Lenarčič, 2013b). For example, in the case of cathepsin B two histidines are located in an insertion that comprises 20 amino acids in the L subdomain referred to as occluding loop. These two residues engage the C-terminus of the peptidic substrate in hydrogen bond/salt bridge contacts, which results in carboxydipeptidase activity by blocking the access of the substrate beyond the S^2′^ binding sites (Figure 2). Owing to the flexibility of the occluding loop and the variable protonation state of the histidine side chains, cathepsin B can act both as exo- and endopeptidase. Typically for papain-like proteases, the endopeptidase specificity of the cysteine cathepsins is mainly governed by the S^2^–P^2^ interactions. Investigations on oligopeptides have revealed that the substrate specificity strongly overlaps and differs only subtly between the individual members (Choe et al., 2006). However, regarding protein substrates, the specificity seems to be more distinct among the different cathepsins (Biniossek et al., 2011) and several protein substrates are selectively cleaved in the presence of other proteins. This selectivity might arise from the fact that the cathepsins, as the majority of proteolytic enzymes, recognize their substrates in an extended conformation that in proteins can only be adopted within flexible loops (Madala et al., 2010). Furthermore, the sterical accessibility of the recognition sequences will certainly determine their substrate properties in addition to the primary structure and conformational properties. The distinctive action on protein substrates enables the specialized roles of the cysteine cathepsins in the molecular wheelwork of crucial cellular processes. In this regard, it is interesting to point out that many cathepsins are able to hydrolyze components of the extracellular matrix, such as various types of collagen, elastin, proteoglycans, and fibronectin. The particular matrix proteins that are subject to cysteine cathepsin-mediated proteolysis have been reviewed in detail recently (Brömme and Wilson, 2011; Fonovič and Turk, 2014a). All cysteine cathepsins are synthesized as preproenzymes. The short N-terminal presequences direct the translated proteins into the endoplasmic reticulum, where this sequence is removed by the action of the signal peptidase. The resulting proenzymes, also known as zymogenes, are catalytically inactive as the proregions (propeptides) block the active sites. Notably, isolated propeptides act as potent inhibitors toward the mature cysteine cathepsins (Fox et al., 1992; Kreusch et al., 2000). The lengths of the proregions vary from 36 amino acids in the case of cathepsin X to 251 amino acids for cathepsin F. The parts of the propeptides which are directly attached to the N-termini of the mature enzymes adopt an extended conformation and block the access to the active sites by binding in reverse orientation compared to the substrates. The propeptides can be removed autocatalytically in the acidic environment of the lysosome or catalyzed by other proteases. In addition to preventing a premature activation of the zymogenes, the proregions act as templates for the folding of the catalytic domains and direct the enzymes into endosomal-lysosomal cell compartments (Wiederanders et al., 2003). Besides the propeptide-catalytic domain interaction the activity of the cysteine cathepsins is strictly regulated by a variety of endogenous proteinaceous inhibitors. Among them, the largest group is represented by the proteins of the cystatin superfamily, which can be subdivided into the actual cystatins and the stefins as well as the kininogens. The cystatins consist of 110–120 amino acids, contain two disulfide bridges and are mainly present outside the cells. In contrast, the stefines are intracellular proteins of similar size without disulfide bonds, whereas the kininogens represent blood plasma proteins with molar masses of 50–120 kDa. In addition to act as cysteine cathepsin inhibitors, the kininogens are implicated in blood pressure regulation as they can be converted to kinines upon limited proteolysis mediated by the kallikrein serine proteases. The cystatin-like proteins inhibit cysteine cathepsins more or less unselectively with inhibition constants in the picomolar range (Turk et al., 2001a). A further group of proteinaceous inhibitors is represented by the thyropins, which are proteins that exhibit homology to thyroglobulin I. Interestingly, the p41 fragment of the MHC II-associated invariant chain has been identified as a selective inhibitor of cathepsin L (Turk et al., 2001b). Serpins, proteinaceous inhibitors which inhibit serine proteases by the formation of covalent complexes, can also act on papain-like cysteine proteases in a similar manner (Schick et al., 1998). Details about the proteinaceous cysteine protease inhibitors are discussed by several review articles, covering both structural as well as physiological aspects (Otto and Schirmeister, 1997; Grzonka et al., 2001; Turk et al., 2002; Rzychon et al., 2004).

Physiological regulation/modulation of cysteine cathepsin activity can furthermore occur in the presence of glycosaminoglycans (Novinec et al., 2014). The phenomenon has been studied in detail for the modulation of cathepsin K's collagenolytic activity by chondroitin sulfate (Li et al., 2002). An X-ray crystal structure of the enzyme in complex with chondroitin-4-sulfate has been solved (Li et al., 2008). Furthermore, it has been demonstrated that both chondroitin-4-sulfate and dermatan sulfate bind to cathepsin K with weak affinity (*K*_*D*_-values 33 and 9.2 μM, respectively) resulting in a decrease of the apparent *K*_*m*_-value for low-molecular-mass substrates (Novinec et al., 2010). In contrast, the hydrolytic activity of cathepsin S is weakly inhibited upon interaction with chondroitin-4-sulfate (Sage et al., 2013). Similar observations have been made with cathepsin B in the presence of heparin and heparin sulfate, while the enzyme experiences stabilization when being complexed with these glycosaminoglycans at slightly alkaline pH (Almeida et al., 2001).

## Inhibitor design and importance of cysteine cathepsins as drug discovery targets

**Figure 4:**
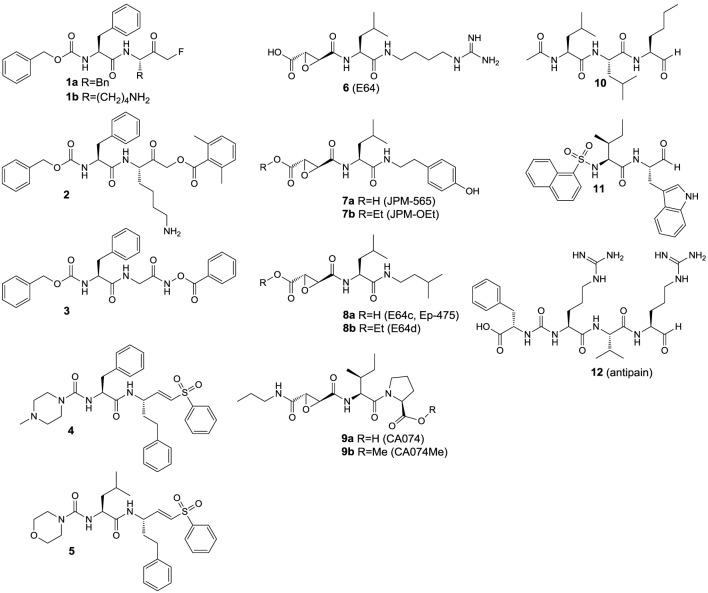
**Inhibitors that were used as experimental tools in studies that are referred to in this article**.

Inhibitors based on small molecules that target papain-like cysteine proteases have contributed substantially to the understanding of the catalytic mechanisms, enzyme-substrate recognition and the biological functions of this class of proteolytic enzymes. Most of the known low molecular weight inhibitors of papain-like proteases are containing electrophilic entities, referred to as warheads, which give rise to irreversible or reversible covalent interactions with the active-site cysteine. The field of small-molecule based inhibitors has been surveyed by several excellent and comprehensive review articles that appeared in the recent years (Otto and Schirmeister, 1997; Leung et al., 2000; Lecaille et al., 2002; Powers et al., 2002; Abbenante and Fairlie, 2005; Frizler et al., 2010). Therefore, only a brief discussion of those inhibitor classes that served as lead structures for the design of imaging probes reviewed in the next section or those which were used as molecular tools for their biological evaluation will be given herein (Figure 4). Compound classes that exert inhibition by irreversible covalent modification of the active-site cysteine residue are peptide-derived halomethylketones, diazoketones, acyloxymethylketones, *O*-acyl hydroxamates, vinyl sulfones and epoxides, among other inhibitor chemotypes. Irreversible inhibitors of cysteine proteases have been reviewed in detail by Powers et al. The following remarks are mainly extracted from this reference and also kinetic data on the inhibition by some of the compounds in Figure 4 can be found therein (Powers et al., 2002). Peptidic chloromethyl ketones have been developed as inactivators of serine proteases but they are also potent cysteine protease inhibitors due to S-alkylation of the active-site cysteine. Their strong intrinsic reactivity results in modification of other biological thiols such as glutathione, even though in slower rates than their interaction with the targeted cysteine proteases. This drawback renders such inhibitors unsuitable for the application in cell-based and animal experiments. In contrast, fluoromethyl ketones such as compounds **1a** and **b** are much less reactive toward thiols, due to the low energy of the carbon-fluorine bond. Despite their low inherent reactivity they are potent inactivators of cysteine proteases and show selectivity over serine proteases. Similarly, diazomethyl ketones can be considered to be selective for cysteine proteases, even though inactivation of serine proteases by this inhibitor chemotype has been reported for some instances. Their reactivity toward thiols of low molecular weight is negligible and some radiotracers have been developed on the basis of this inhibitor class (see Section ^125^I-labeled Compounds). Acyloxymethyl ketones (AOMK's; see compound **2** in Figure 4 for example) have been developed with the intention to create deactivated versions of chloromethyl ketones. This inhibitor chemotype has been shown to inactivate cysteine proteases selectively over other classes of proteolytic enzymes. In addition to the other reactive methyl ketones, they provide the opportunity of kinetic tuning upon varying the acyloxy leaving group. However, their potential susceptibility toward esterase-catalyzed degradation might compromise their applicability *in vivo* (Verdoes et al., 2013). Formal isoelectronic replacement of the methylene group in the latter inhibitor class leads to *O*-acyl hydroxamates, as represented by compound **3**. This chemotype forms covalent complexes with the active-site cysteine in analogy to acyloxymethyl ketones. In contrast, peptide-derived *O*-acyl hydroxamates are capable to inactivate serine proteases, even though their mechanism of interaction may differ between these two classes of proteases (Brömme and Demuth, 1994). Mechanistically related to the aforementioned inhibitor classes are peptide-derived epoxides, among which the epoxysuccinyl peptides constitute the most important subclass. Differently to reactive methyl ketones, no nucleofuge is leaving the enzyme-inhibitor complex, as the attack of the active-site thiol results in epoxide opening. Their prototype is represented by the fungal secondary metabolite E64 (**6**), which has been shown to inactivate a broad spectrum of papain-like cysteine proteases. Replacement of the guanidinobutyl moiety by hydroxyphenyl and isobutyl led to JPM-565 (**7a**) and E64c (**8a**), respectively, without significant changes in the inhibitory potential. Replacement of the leucine residue in E64 (**6**) by isoleucine, C-terminal extension by proline and amidation of the carboxylic group results in CA074 (**9a**; Figure 4), which inactivates cathepsin B selectively over other cysteine cathepsins. Notably, epoxysuccinyl peptides can bind to the active site in the same or inverse orientation compared to the substrate binding mode. Esterification of the carboxylic groups in JPM-565, CA074, and E64 with simple alcohols renders these highly polar molecules membrane permeable (compounds **7b**, **8b**, and **9b** in Figure 4). Despite the actual inhibitors can be released by the action of cellular esterases, the corresponding “prodrugs” still bear inhibitory potential but altered selectivity profiles compared to their “active” progenies, and cysteine cathepsin inactivation may occur faster than ester hydrolysis (Bogyo et al., 2000). Therefore, the results of cell-based experiments in which these esterified epoxysuccinyl peptides are used for pharmacological inhibition should be interpreted with care in terms of selectivity toward individual cysteine cathepsins. E64d (**8b**) has been demonstrated to be bioavailable upon oral administration. The spontaneous reactivity of epoxide inhibitors toward thiols has been shown to be low. A couple of radiotracers have been developed starting from epoxysuccinyl peptides (see Section ^125^I-labeled Compounds). Besides epoxysuccinyl peptides, peptidic inhibitors with epoxide warheads have been also created by replacing formally the terminal carboxylic group of N-terminal cleavage products by an oxirane ring, resulting in *N*-peptidyl-α-aminoalkyl epoxides. They interact with the active site by epoxide opening upon attack of the active-site sulfhydryl group at the methylene carbon. Concerning the two possible configurations at the methine carbon, the *erythro* diastereomers are more active than their *threo*-configured counterparts. In agreement with the altered electronic situation of the oxirane moiety, *N*-peptidyl-α-aminoalkyl epoxides are less potent inactivators of cysteine proteases compared to epoxysuccinyl derivatives. A tritium-labeled *N*-peptidyl-α-aminoalkyl epoxide has been reported (see Section Radiotracers Based on Fluorine-18, Radiocarbon Isotopes, and Tritium; and compound [^3^H]**39** in **Figure 11**) (Albeck, 2000; Powers et al., 2002). Vinyl sulfones are cysteine-reactive moieties that give rise to nucleophilic addition instead of substitution. Their incorporation at the C-terminus of small peptides can lead to highly potent inactivators of cysteine proteases such as compounds **4** and **5** with limited inherent thiol reactivity. Compound **5** has been used as lead structure for radiotracer design (see Section ^125^I-labeled Compounds). Despite vinyl sulfones inactivate cysteine proteases selectively over serine proteases, the introduction of three leucine residues into position P^1^–P^3^ renders them capable of ether formation with the active-site threonine residue in proteasomes.

In contrast to vinyl sulfone warheads for which attack by the active-site thiol is irreversible, addition to some electrophilic functional groups can be reversible, which allows the enzyme-inhibitor complex to dissociate. This holds true for peptide-derived aldehydes and ketones as well as nitriles, which give rise to the formation of thiohemiacetals (Otto and Schirmeister, 1997; Shokhen et al., 2011) and thioimidates (Frizler et al., 2010), respectively. Peptide aldehydes such as compounds **10–12** are highly potent inhibitors for cysteine proteases (Lecaille et al., 2002). They potentially can also inhibit serine proteases by interactions analogous to cysteine proteases (Ruiz-Gómez et al., 2009). For example, the naturally occurring aldehyde antipain (**12**) potently inhibits serine proteases such as trypsin and thrombin, in addition to its inhibitory activity toward a variety of cysteine proteases^2^. Amino acid and peptide-derived aldehydes bear furthermore the potential to inhibit metallopeptidases, because the oxygen atoms of the hydrate forms can mediate coordinative interactions with the Zn^2+^ ion in the active site (Sträter and Lipscomb, 1995). In contrast, cyano groups are less reactive electrophiles, which is the reason why peptide-derived nitriles can interact quite selectively with cysteine over serine proteases (Frizler et al., 2010). Exceptions are known for the serine protease dipeptidyl peptidase IV, for which dipeptide nitrile inhibitors have been developed as the antidiabetic drugs vildagliptin and saxagliptin (Nabeno et al., 2013).

**Figure 5:**
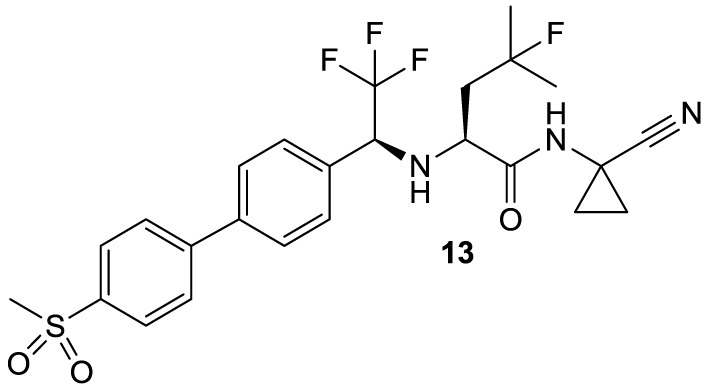
**Odanacatib, probably the first cysteine protease inhibitor that will enter the drug market**.

Partly due to the employment of the aforementioned inhibitors as experimental tools, the perception about the biological functions of cysteine proteases, especially those of the cathepsins, has changed dramatically over the last 20 years and until today the involvement in a plethora of individual cellular processes has been revealed for almost every individual cathepsin (Brix et al., 2008; Reiser et al., 2010). An increased activity of the cysteine cathepsins, which can result from increased expression or a reduced level of their endogenous protein inhibitors, has implications in several pathologies (Katunuma, 2011). Consequently, the cysteine cathepsins became highly attractive targets to develop drugs for the treatment of systemic human diseases (Turk et al., 2003). These diseases include autoimmune and allergic disorders (Reiser et al., 2010; Löser, 2011; Perišic Nanut et al., 2014), cardiovascular diseases, osteoporosis and cancer. In light of the pivotal role of cathepsin K in the degradation of osseous collagen, inhibitors directed against this protease have been developed to treat osteoporosis (Novinec and Lenarčič, 2013a). These efforts will probably lead to the first cathepsin inhibitor, named odanacatib, that can be launched to the drug market (**13**; Figure 5). Odanacatib is a dipeptide-derived nitrile which targets selectively cathepsin K. This compound is currently in phase III clinical studies for osteoporosis treatment, e.g., the Long-Term Odanacatib Fracture Trial (LOFT) (Bone et al., 2015), and in various phase II and III clinical trials targeting metastatic bone disease in advanced-stage breast and prostate cancer (Onishi et al., 2010; Sturge et al., 2011)^3^^,^^4^^,^^5^. Its discovery has been summarized in Gauthier et al. (2008). The most salient structural features of odanacatib are the replacement of the P^3^ carbonyl oxygen by a trifluoromethyl group, fluorination at C_γ_ in the P^2^ leucine side chain and a cyclopropyl group that integrates the C_α_ atom in P^1^. The advantages of peptide bond replacement by trifluoroethylamines have been outlined in Bigotti et al. (2009). Side-chain fluorination intended to block oxidative metabolism at the methine group and introduction of the cyclopropyl group stabilizes the P^2^-P^1^ amide against metabolic hydrolysis (Gauthier et al., 2008).

Furthermore, clinical studies associated with targeting cysteine cathepsins by small organic molecules have been reported in the context of cathepsin S inhibitors for the treatment of rheumatoid arthritis, psoriasis and neuropathic pain (Fonovič and Turk, 2014b). Generally, cathepsin S inhibition has a high potential to treat autoimmune-related disorders due to its pivotal role in MHC II-dependent antigen presentation (Gupta et al., 2008). Dysregulated antigen presentation in keratinocytes has been linked to the pathogenesis of psoriasis (Schönefuß et al., 2010). Moreover, cathepsin S has been demonstrated to elicit pruritus by activating the protease-activated receptors PAR-2 and PAR-4 in cutaneous nerve fibers (Reddy et al., 2010). These results contribute to the rationale for developing cathepsin S inhibitors as antipsoriatic agents. Treatment of neuropathic pain by inhibiting cathepsin S is mainly deduced from the enzyme's ability to liberate the CX_3_C chemokine fractalkine from a precursor bound to the surface of dorsal horn neurons, which triggers inflammatory signaling in microglial cells (Clark and Malcangio, 2012).

## Functions of cysteine cathepsins in tumor progression and their influence on therapy response

For neoplastic diseases, accumulating evidence confirms the crucial role of several cysteine cathepsins in the progression, invasion and metastasis of solid tumors. They furthermore seem to be influencing the response of tumor cells to ionizing radiation and cytostatic agents (Gocheva and Joyce, 2007; Palermo and Joyce, 2008; Lankelma et al., 2010; Mason and Joyce, 2011; Reinheckel et al., 2012; Kos et al., 2014). Despite increasing evidence for pro-tumorigenic functions of cysteine cathepsins, individual roles for individual members are difficult to define, which is partly due to the heterogeneous nature of tumor biology. Generally, the fatality of neoplastic diseases mainly arises due to the dissemination of primary tumor cells from their initial location to colonize distant organs. Even though the range of target tissues varies among different tumor entities, neoplasms of the same origin often share the organ pattern of metastasis. For example, breast and lung adenocarcinomas tend to metastasise to organs such as bone, lung, liver and brain (Nguyen et al., 2009). For being able to disseminate from their original location, tumor cells need to acquire an invasive phenotype. Therefore, they have to undergo a process of de-differentiation called epithelial-to-mesenchymal transition (EMT). During this process, which is initiated by certain growth factors, cells undergo a variety of functional and morphological changes such as impairment of cell–cell contacts, loss of apical-basolateral polarity, increased cellular motility and enhanced extracellular proteolysis (Thiery, 2002; Joyce and Pollard, 2009; Box et al., 2010).

Regarding the EMT hallmark of increased extracellular proteolysis, the cathepsins are considered to be key players in this process, in addition to the proteolytic activities that are exhibited by the matrix metalloproteinases, the uPA/plasmin system and the aspartic proteases cathepsins D and E, and, furthermore, serine proteases of the kallikrein family (Schmitt et al., 1992; Borgoño and Diamandis 2004; Lee et al., 2004; Affara and Coussens, 2008; Fröhlich, 2010; Sevenich and Joyce, 2014; Theocharis et al., 2014). Partial degradation of the extracellular matrix results in its remodeling, which creates niches and trails for cell migration and uncovers binding epitopes at matrix proteins that can be recognized by receptors involved in cell–matrix interactions such as integrins (Friedl and Alexander, 2011). The functional contribution of the cysteine cathepsins to tumor invasion and metastasis is manifold. While the potential implication in tumor progression has been proposed for all of the 11 cysteine cathepsins (Mohamed and Sloane, 2006), the most striking evidence exists for the cathepsins B, L, S, K, and X (Joyce et al., 2004).

Besides the degradation of matrix proteins, cysteine cathepsins are capable of shedding cell surface molecules that are important for cell adhesion such as cadherin E. This cell surface protein mediates cell-cell contacts by homophilic interactions and the attenuation of its expression on malignant cells correlates with a more invasive tumor phenotype. It has been demonstrated that cadherin E can be specifically cleaved by the cathepsins B, L and S and thereby removed from the cell surface by these proteases (Gocheva et al., 2006).

Not only extracellular but also intracellular protein hydrolysis catalyzed by cathepsins can contribute to tumor progression. This has been concluded from experiments in which inhibition of intracellular cathepsins by E64d (**8b**) reduces the TGFβ-1 stimulated invasive capacity of the malignant epithelial cell lines iPL32 and A549. Because cell migration was impeded even one day after treatment with **8b** had been stopped, a direct inhibition of extracellular proteolysis during the invasion assay is unlikely to explain this effect. Furthermore, the effect was accompanied by the accumulation of enlarged lysosomes in these cells, which indicates lysosomal protein storage upon inhibition of cysteine cathepsins (Kern et al., 2015). The work by Kern et al. confirms similar results that have been obtained by exposing neuroblastoma cells to compound dideiodo-**26b** (see **Figure 10** for structure), a diazoketone which is a potent inactivator of the cathepsin B and L (Colella et al., 2010; Cartledge et al., 2013). In line with these results, exposure of tumor-associated macrophages to acyloxymethyl ketone **2**, which is capable of inactivating the cathepsins S, L and B, causes oxidative stress and subsequent apoptosis. These effects were attributed to the influence of **2** on intracellular proteolysis. The impact of **2** on tumor growth *in vivo* has been investigated in the syngeneic murine 4T1 model. Compound **2** was administered in a dose of 40 mg/kg once a day for 3 days. At day 4 the tumors of the sacrificed mice were investigated for apoptosis markers by FACS analysis, which revealed increased programmed cell death throughout the tumor. Accordingly, the tumor size was significantly reduced compared to the untreated control. Of note, **2** did not affect the growth of 4T1 tumor cells *in vitro*, which indicates the importance of tumor-associated macrophages for cancer growth (Salpeter et al., 2015).

The member of the cysteine cathepsin family that has been most intensively discussed in the context of cancer is cathepsin B (Sloane et al., 2005; Gondi and Rao, 2013; Aggarwal and Sloane, 2014; Kos et al., 2014; Lampe and Gondi, 2014). The enzyme can be located at the surface of invasive tumor cells in areas such as focal adhesions and invadopodia where it associates with the peripheral membrane protein annexin A2. It can be also present in membrane invaginations rich in cholesterol and sphingolipids called caveolae by forming complexes with the protein caveolin-1. Membrane association of cathepsin B seems to support proteolytic processing of extracellular substrates such as plasminogen and tissue-type plasminogen activator (tPA), tenascin C and type I and IV collagens (Werner et al., 2012). Elevated activity of cathepsin B has been shown to increase the malignancy of various neoplasia such as breast cancer (Withana et al., 2012), glioma (Gole et al., 2009), and melanoma (Matarrese et al., 2010).

The involvement of cathepsin L in tumor progression is less evident than for cathepsin B but its contribution to the invasive and metastatic potential of malignant cells such as oncogenically transformed fibroblasts and melanoma cells has been demonstrated (Ravanko et al., 2004; Rousselet et al., 2004). Furthermore, higher levels of cathepsin L in primary malignant melanomas have been correlated to poor prognosis (Štabuc et al., 2005). Taken together, these results render this protease a potential target in anti-tumor therapy (Lankelma et al., 2010).

The functions of cathepsin S in neoplastic progression are manifold (Chang et al., 2007). Recent results provide evidence that secretion of this cysteine protease from tumor-associated macrophages and endothelial cells is important for tumor angiogenesis, which is in agreement with findings from earlier studies (Small et al., 2013). Nidogen-1, a constituent of the basement membrane, has been shown to be subject of limited proteolysis catalyzed by cathepsin S (Sage et al., 2012). This finding provides potential evidence that this enzyme might be actively involved in tumor invasion. Very recently, the function of cathepsin S in brain metastasis of primary mamma tumors has been investigated in detail (Sevenich et al., 2014). In this study, both genetic ablation and pharmacological inhibition of cathepsin S with a compound of undisclosed structure has been demonstrated to result in suppression of the metastatic spread of human MDA-MB-231 breast cancer cells to the brain in a mouse model. In particular, cathepsin S facilitates blood-brain barrier passage of tumor cells by specific proteolytic cleavage of the junctional adhesion molecule JAM-B. Interestingly, cathepsin S is contributed by both tumor and stroma cells with higher expression of tumor-derived cathepsin S in early brain metastases compared to late stage while the expression pattern of stromal cathepsin S was inversed. These results highlight the potential of this cysteine protease to drive colonization of distant organs during tumor metastasis.

The tumor-promoting functions of cathepsin K have been mainly discussed in the context of bone metastasis (Podgorski et al., 2007). This issue was briefly mentioned in the previous section. Apart from its well-confirmed involvement in metastatic bone disease, cathepsin K seems to have a more general role in tumor progression. For example, immunoreactivity against this cysteine cathepsin correlates with increased invasive potential of lung adenocarcinoma (Rapa et al., 2006).

Accumulating evidence indicates that cathepsin X is a key protease in tumor invasion. Unique among the cysteine cathepsins, the enzyme exhibits a strict carboxymonopeptidase and—dipeptidase activity without acting as endopeptidase (Nägler et al., 1999; Klemenčič et al., 2000). However, the action of cathepsin X in tumor invasion seems to be mainly independent of its catalytic properties. This is attributed to the presence of an RGD motif in its proregion in a conformation very similar to cyclic RGD-pentapeptides. This enables procathepsin X to interact with the integrin subtype α_*v*_β_3_ (Lechner et al., 2006). It has been shown that cathepsin X acts as a promoter of T-lymphocyte migration along ECM-mimicking Matrigel, which is partly independent of its proteolytic activity due to stimulation of integrin signaling (Jevnikar et al., 2008). Accordingly, detailed investigations in a mouse model of pancreatic neuroendocrine tumors have revealed that cathepsin X expressed by both tumor cells and tumor-associated macrophages drives malignancy via integrin signaling without involving its proteolytic activity. Interestingly, procathepsin X secreted from tumor-associated macrophages was capable of stimulating the invasive potential of the cancer cells while their proliferation could only be stimulated by procathepsin X expressed by the cancer cells themselves. This phenomenon probably originates from interactions between procathepsin X and the intracellular domain of the integrin β chain (Akkari et al., 2014).

The findings mentioned above highlight the manifold functions of cysteine cathepsins in general tumor development. In the following subsection we will discuss their role in the response of tumors to cytostatic therapies.

### Considerations on cysteine cathepsin inhibitors as adjuvant radiosensitizers and chemosensitizers

The improved methods for prevention and early detection of cancer, as well as recent advances in cancer treatment, have served to substantially decrease cancer-related mortality. The most obvious advances have been made by combining improved surgical techniques with cytotoxic ionizing radiation and chemotherapy. Although radiation- and drug-based therapeutic modalities provide large therapeutic benefit for cancer patients, physicians are concerned with their adverse side effects. Most prominent is the risk that patients may develop neoplastic disorders as a consequence of radio- or chemotherapy. But also non-neoplastic disorders, such as development of organ failure, fibrosis or atherosclerosis, are serious health consequences of cancer therapy. Moreover, development of tumor resistance to therapy will affect the overall individual outcome of each patient. One may mitigate these risks by adjusting doses and frequency of treatment, but this often leads to lower therapy effectiveness and, consequently, insufficient tumor control. Hence, there is a growing area for new drug development in the field of supportive or facilitating agents used in adjuvant therapies, which are intended to intensify tumor-targeted effects and/or to avert or minimize treatment-limiting toxicity to normal tissue.

As mentioned above there is experimental and preclinical evidence that various cysteine cathepsins, particularly, the cathepsins B, L, and S, are important mediators of cancer therapy response and outcome in various cancer entities. In this regard, the modulation of processes that induce cancer cell senescence, apoptosis or necrosis by cysteine cathepsins in an activating or inhibiting manner seems to be of particular importance (Česen et al., 2012; Aits and Jäättelä, 2013). Logically, targeted selective or multiple inhibitors of cysteine cathepsin activity have been proposed as promising adjuvant therapeutics forcing cancer cell death in combined therapeutic regimen (Turk et al., 2012).

This gains importance for internal radionuclide-based or external radiation therapy as well as for chemotherapeutic approaches. Exemplarily, Seo et al. (2009) demonstrated that irradiation with γ-rays induced overexpression of cathepsin S both on RNA and protein level in human breast cancer cells in a dose-dependent manner. This modulation of expression is mediated by reactive oxygen species (ROS)-interferon-γ (IFN-γ) pathways. These authors also found the expression of other cathepsins, in detail cathepsin D, cathepsin L and cathepsin B, to be induced as a response to ionizing radiation. However, in their study they focused in particular on cathepsin S functions. Cathepsin S promoter activity is mediated by the interferon regulatory factor-1 (IRF1), which binds to a single interferon-stimulated response element (ISRE) site. This ISRE site is located 100 bp upstream from the transcriptional start site of the cathepsin S promoter. In an earlier investigation Storm van's Gravesande et al. (2002) showed that IFN-γ treatment enhanced the affinity of IRF1 to cathepsin S IRSE oligonucleotides. Consistently, Seo et al. demonstrated that radiation-induced IFN-γ induces IRF1 and, subsequently, increases cathepsin S promoter activity. As a consequence, high expression of cathepsin S enhanced the radioresistance of tumor cells and transfection with cathepsin S-siRNA blocked this effect. These results are in line with the observation of high cathepsin S expression in other radioresistent tumors, e.g., glioblastoma (Flannery et al., 2003, 2006). Notably, radiation response of cathepsin S also occurred in non-tumorigenic cells (Seo et al., 2009). The role of cathepsin S in radiation-induced adverse effects in normal tissues is still unclear. However, it can be hypothesized that targeted inhibition of cathepsin S as adjuvant therapy possibly results in both radiosensitization of tumors and protection of normal tissues. More recently, Malla et al. demonstrated that cathepsin B increased with X-ray radiation in a dose-dependent manner in human glioblastoma cells, thus likewise contributing to radioresistance. Transfection of these cells with cathepsin B-siRNA resulted in downregulation of the enzyme and higher radiosensitivity. Of interest, in this case cathepsin B downregulation contributed to increased apoptosis rate in the irradiated glioblastoma cells (Malla et al., 2012). Furthermore, genetic predisposition of tumors can result in cysteine cathepsin overexpression, thereby contributing to intrinsic radioresistance or resistance to genotoxic drugs. In this regard, Grotsky et al. (2013) recently demonstrated that loss of the tumor suppressor BRCA1 activates cathepsin L-mediated degradation of the DNA repair factor 53BP1 in human breast tumor cells. In this study depletion or inhibition of cathepsin L with vitamin D or the broad-spectrum cysteine cathepsin inhibitor E64 (**6**) stabilized 53BP1, which results in higher genomic instability in response to both radiation and genotoxic drugs. Consistent with the *in vitro* experiments, analysis of human breast tumor samples identified cathepsin L as a biomarker which inversely correlates with 53BP1 (Grotsky et al., 2013). This observation is potentially of predictive value for therapy response of individual patients.

These and also other observations contribute to recent findings which indicate a potential antiapoptotic function of cysteine cathepsins in tumor cells. In this regard, enhanced expression of cathepsin B has been shown to rescue rat pheochromocytoma cells from apoptosis induced by serum deprivation (Shibata et al., 1998). On the other hand, downregulation of cathepsin B using antisense phosphorothioate oligonucleotides induced apotosis in these cells (Isahara et al., 1999). Moreover, chemical inhibition of cathepsin B using the selective dipeptide-derived *O*-benzoyl hydroxamate **3** in various highly genotoxic drug- and radiation-resistant human tumor cells likewise induced apoptosis, which is also contributing to the hypothesis on antiapoptotic survival-promoting functions of cysteine cathepsins in human cancer (Zhu and Uckun, 2000). Accordingly, a study published by Wadhawan et al. (2014) explains that E64 significantly inhibits filarial cathepsin B activity followed by generation of oxidative stress and induction of a mitochondrial mediated apoptosis in filarial parasites (*Setaria cervi*). These results suggest that antiapoptotic function of cysteine cathepsins is not limited to human tissue.

The findings mentioned above are in apparent contradiction with reports suggesting cysteine cathepsins being mediators of lysosomal-mediated cell death (Colletti et al., 2012). Exemplarily, Gores and coworkers demonstrated that cathepsin B contributes to bile salt-induced apoptosis in rat hepatocytes and rat hepatoma cells. Both chemical inhibition of cathepsin B using the highly selective cathepsin B inhibitor CA074 (**9a**) and expression of cystatin A prevented cathepsin B activation and apoptosis during treatment with glycochenodeoxycholate, a toxic bile salt (Roberts et al., 1997). However, in the context of the investigation of these authors, the used hepatocyte-derived cell line McNtcp.24, which is stably transfected with a bile salt transporter, is more likely a model of hepatocyte injury than of liver cancer. Consequently, these specific pro-apoptotic functions of cathepsin B could be interpreted as part of normal tissue homeostasis. This interpretation is consistent with observations of the contribution of another cysteine cathepsin, cathepsin L, to ultraviolet-induced apoptosis in epidermal keratinocytes (Welss et al., 2003). On the other hand, the observation of increased glioblastoma cell death in cells transfected with parvovirus H-1 showing an accumulation of cathepsins B and L in the cytosol or reduced levels of cystatin B and C, suggests pro-apoptotic function of cysteine cathepsins also in tumor cells (Di Piazza et al., 2007). Taken together, it is important to note that the roles of cysteine cathepsins in cell senescence, lysosomal-mediated cell death/apoptosis, and necrosis are still not fully understood. In this regard, various factors that could affect the suitability of cysteine cathepsin targeting in adjuvant therapeutic settings must be considered in advance. Particularly, these factors comprise intracellular localization, such as lysosomal or cytosolic and extracellular localization, the tissue type as well as the tumor entity, localization, and microenvironmental influences.

However, based on the considerations above, inhibition of cysteine cathepsins also is hypothesized to be a strategy for chemosensitization or chemopotentiation in a standard or targeted chemotherapy regimen. Exemplarily, a combination of doxorubicin and the fluoromethylketone-based cathepsin L inhibitor **1a** enabled the induction of senescence in various murine and human drug-resistent cancer cell lines (Zheng et al., 2004). More evidence for chemosensitizing effects due to cathepsin inhibition has been provided by the same group more recently by showing that the cathepsin L-selective aldehyde inhibitor iCL (compound **11**) led to a reversal of resistance to doxorubicin in human neuroblastoma and osteosarcoma cells *in vitro* and in nude mice xenografted with doxorubicin-resistant human neuroblastoma cells *in vivo* (Zheng et al., 2009). With respect to the mechanism of the chemosensitizing action of this inhibitor, Zheng and coworkers demonstrated that its use stabilizes and enhances the availability of cytoplasmic and nuclear proteinaceous drug targets including estrogen receptor-alpha, Bcr-Abl, topoisomerase-IIα, histone deacetylase 1, and the androgen receptor. Furthermore, these authors demonstrated that compound **11** also enhanced the cellular response to tamoxifen, etoposide, imatinib, vinblastine, and trichostatin A (Zheng et al., 2009). Of note, this investigation revealed no chemosensitizing action of **11** on cisplatin. In contrast, there is experimental evidence that inhibition of cysteine cathepsins also seems to affect in a positive manner platinum resistance mechanisms. In this regard, Jacquemont et al. (2012) identified various compounds that were able to sensitize ovarian cancer cells to cisplatin. Among them, the tripeptide aldehyde **10** and the selective irreversible cell-permeable inhibitor CA074Me (**9b**) substantially synergized with cisplatin. Of note, the platinum resistance mechanisms investigated was monoubiquitination and nuclear foci formation of FANCD2, a crucial step in the so-called Fanconi anemia pathway. In addition to the *in vitro* data, a combination regimen of “chemo-switch” cyclophosphamide, a DNA-alkylating agent, and cysteine cathepsin inhibition using the cell-permeable broad-spectrum inhibitor JPM-OEt (**7b**) was demonstrated to be very effective in preclinical trials, as it reduced the tumor burden and extended the survival in a RIP1-Tag2 mouse model of pancreatic islet cell carcinogenesis (Bell-McGuinn et al., 2007). Conversely, combined treatment of mouse lymphosarcoma using cyclophosphamide and E64c (**8a**) stimulated tumor growth and reduced the antitumor effect of cyclophosphamide (Zhanaeva et al., 2005). These again contradictory results contribute to a critical analysis of the benefit of combining cysteine cathepsin inhibitors with chemotherapeutics. However, such adjuvant therapeutic settings targeting cysteine cathepsins will not only affect tumor cells but also stromal cells and microenvironment-supplied factors. In this regard, stromal cells such as tumor-associated macrophages become an important target. Tumor-associated macrophages are abundant suppliers of cysteine proteases, which are important for enhancement of tumor growth and invasion (Small et al., 2013; Bengsch et al., 2014). Furthermore, macrophages provide survival signals to tumor cells in a cathepsin-dependent manner, which abrogates tumor cell death induced by various stimuli. Such “chemoprotective” effects of cathepsins were identified for taxol, etoposide, and doxorubicin. Logically, inhibition of cathepsin activity is sufficient to minimize or abrogate this protective effect, as demonstrated in breast cancer, for example (Shree et al., 2011). Of interest, therapeutical approaches that exert effects on activity and localization of cysteine cathepsins in an indirect manner also may result in chemosensitization. This has been demonstrated very recently in human hepatocellular carcinoma cells for which suppression of CD47 by a morpholino approach exerted a chemosensitization effect through blockade of cathepsin S/protease-activated receptor 2 (PAR2) signaling (Lee et al., 2014).

On the other hand, inhibition of cysteine cathepsins in cancer patients may not always be desirable and will strongly depend on the type of chemotherapeutic drug. Exemplarily, to overcome dose-limiting side effects of doxorubicin-like cardiotoxicity, an intensive effort has been undertaken to develop promising doxorubicin peptide prodrugs targeted to, e.g., cysteine cathepsins that are specifically activated at the tumor site (see Section Substrate-based Probes). The addressed cysteine cathepsins, particularly, cathepsin B, then catalyze the activation of these prodrugs, and hence, the regulation of this enzyme by antitumor agents could influence the efficacy of these peptide prodrugs (Bien et al., 2004; Zhong et al., 2013).

Potential adverse or side effects that may accompany inhibition of cysteine cathepsins are more generally to be regarded as limiting criteria. Exemplarily, inhibition of cathepsin B and L results in lysosomal dysfunction and consequent cell death in pancreatic β-cells (Jung et al., 2015), but it is beyond the scope of this review to discuss this in more detail.

## Recent trends in the development of cysteine cathepsin-targeting imaging probes and related molecular tools

The increasing insight into the functions of the cathepsins has also raised interest to follow their actions in living systems by molecular imaging and both fields have mutually stimulated each other. The most relevant imaging modalities that can provide information on the molecular level are optical imaging (OI), single photon emission computed tomography (SPECT), positron emission tomography (PET) and magnetic resonance imaging (MRI) (Quillard et al., 2011; James and Gambhir, 2012; Cunha et al., 2014). The principles of optical imaging will be briefly outlined below, those of SPECT and PET in Section Radiotracers.

Because MRI relies on the phenomenon of nuclear magnetic resonance (NMR), it bears a great potential to obtain biochemical information. One approach to generate molecular contrast in MRI is based on chemical exchange saturation transfer (CEST). This method relies on proton exchange between acidic or basic functionalities and bulk water. Provided that the rate constant of exchange does not exceed the difference in the resonance frequencies of the chemical entity and water, presaturation of the respective protons in the solute by an appropriate radiofrequency pulse will result in saturation transfer to bulk water (Woods et al., 2006; van Zijl and Yadav, 2011). In consequence, the NMR signal of the water protons will be attenuated and thus negative contrast is generated. Very recently, this principle has been applied to image the tumor-associated cathepsin B activity in a rat brain tumor model using poly-L-glutamate as contrast-generating agent (Haris et al., 2014). This syngeneic orthotopic tumor model is derived from 9L rat gliosarcoma cells, which express active cathepsin B. Because hydrolysis of poly-L-glutamate catalyzed by cathepsin B releases exchangeable amine protons, the CEST contrast will increase upon enzymatic action. Ninety minutes after injection of poly-L-glutamate the CEST signal in the tumor region increased by 19%. Despite the potential to detect chemical changes and high spatial resolution, MRI lacks sensitivity compared to the other imaging modalities. For example, a dose of poly-L-glutamate as high as 160 mg/kg had to be injected into rats for CEST imaging of cathepsin B.

In general, the field of molecular imaging depends on the development of compounds consisting of moieties that can generate detectable signals in addition to the entity that mediates biological targeting. Such molecular probes are not restricted to be used in whole-animal imaging but are also valuable tools for *in vitro* and *in cellulo* investigations.

### Fluorescent probes

OI relies on the detection of low-energy photons that can be emitted by processes of fluorescence, chemiluminescence and bioluminescence and, furthermore, by Čerenkov radiation (see Section Radiotracers). Concerning OI of cysteine cathepsins, the reported probes are based on fluorescently labeled substrates and inhibitors. The instrumental setup for fluorescence imaging basically consists of a light source for illumination and a charged coupled device (CCD) camera for detection of the emitted light. Therefore, the required instrumentation is rather inexpensive compared to other imaging modalities. Because the penetration depth of visible light in tissue is limited to only a few centimeters, whole-body OI is restricted to small animals such as mice and rats. Penetration depths are highest for fluorophores that emit photons in the near-infrared region (650–900 nm) of the electromagnetic spectrum. Concerning clinical translation, issues related to penetration depth can be circumvented by applying endoscopic imaging systems (James and Gambhir, 2012). Generally, the advancement in the field of OI is dependent on the engineering of fluorophores with favorable photophysical properties and the development of methods for their chemical conjugation (Kim and Park, 2010; Grimm et al., 2013).

Because cysteine cathepsin-targeting fluorescent probes are covered by other review articles, only recently reported molecular probes of this category will be discussed herein (Blum, 2008; Edgington et al., 2011; Hu et al., 2014b; Sanman and Bogyo, 2014).

#### Activity-based probes

Particular attention has been devoted to the development of probes based on inhibitors which form stable covalent bonds with the active-site cysteine. These molecular probes are referred to as activity-based probes (ABPs) (Schmidinger et al., 2006; Paulick and Bogyo, 2008; Willems et al., 2014). Besides the electrophilic warhead that enables covalent targeting of the active site, these probes consist of a tag containing the reporter group, which allows the detection of the enzyme-inhibitor complex, and a linker. The linker not only connects the warhead with the reporter tag but also confers selectivity by giving rise to non-covalent interactions in the enzyme's binding pockets. Apart from fluorophores that enable fluorescence imaging, reporter groups may be built of affinity tags such as biotin, radionuclide-containing groups (see Section Radiotracers) or moieties modified with non-abundant stable isotopes (Sadaghiani et al., 2007; Serim et al., 2012).

**Figure 6:**
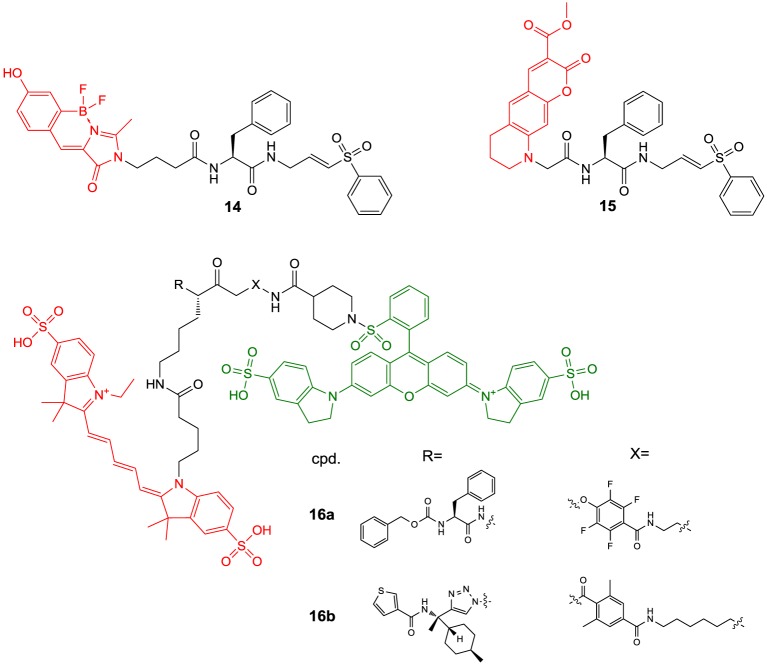
**Fluorogenic ABPs targeting cysteine cathepsins**. Emitting fluorophoric moieties are shown in red, chromophoric moieties that act as quencher are highlighted in green.

More than for *in vivo* imaging, ABPs have been used to detect active proteases in cell lysates by incubating the crude protein mixtures with the labeled probes and subsequent electrophoretic separation followed by label-specific detection. This approach is called activity-based or chemical proteomics and offers the advantage of coupling the detection signal to enzyme activity and therefore provides more accurate information about the functions of the enzyme of interest in the biological process to be studied (Fonovič and Bogyo, 2008; Deu et al., 2012). A recently developed ABP to target cathepsin K is compound **14** (Figure 6) (Frizler et al., 2013). This probe is based on the vinyl sulfone warhead and equipped with an innovative tricyclic luminescent group which represents a conformationally restricted analog of the 4-(4-hydroxybenzylidene)-1*H*-imidazol-5(4*H*)-one fluorophore in green fluorescent protein (Baranov et al., 2012). Compound **14** proved to be an irreversible inactivator of the cathepsins S, K, L, and B with a slight selectivity toward S and K over L and B, while it showed the strongest potency against cathepsin S. Its suitability to visualize cathepsin K *ex vivo* by in-gel fluorescence imaging after electrophoretic separation of the cathepsin K-**14** complex has been demonstrated. Labeling of cathepsin K by **14** could be prevented upon preincubation with a reversible azadipeptide nitrile inhibitor that is highly selective for this cathepsin. The probe enabled the detection of external cathepsin K among the proteins present in HEK cell lysate. A linear correlation between the fluorescence intensity of the electrophoretic spots and the amount of enzyme was observed over a range of 17–280 ng of cathepsin K and the detection limit was found to be superior to Western blot. The ABP **15** is of very similar design as **14** (Mertens et al., 2014). In contrast, it is equipped with an alternative fluorophore on the basis of a novel coumarin-tetrahydroquinoline hybrid. This fluorophore can be considered as a conformationally locked 7-*N*,*N*-diethylaminocoumarin and shows improved photophysical properties over its non-rigidified counterpart such as bathochromically shifted absorption and emission maxima (Frizler et al., 2012; Mertens et al., 2014). Among the cathepsins S, K, L, and B, inactivation by **15** was strongest for cathepsin S with approximately threefold increased *k*_inact_/*K*_I_-values for this cathepsin compared to compound **14**. Probe **15** is more than 50-fold less potent for cathepsin K than for S. This result has been explained on the basis of docking studies, which revealed that the coumarin-tetrahydroquinoline moiety of **15** partially occupies the S^3^ pocket of cathepsin S and its position is probably stabilized by hydrogen bond interactions between its two carbonyl groups and the side chain of Arg141 and the backbone NH of Val162. This aptly illustrates that also the fluorophore may interact with the enzyme and thus can contribute to the selectivity of the probe. Compound **15** was evaluated for in-gel detection of cathepsin S in a similar fashion as **14** for cathepsin K. The fluorescent cathepsin S-**15** complex enabled to visualize enzyme amounts as low as 0.5 ng. Preincubation of cathepsin S with the broad-spectrum cysteine protease inhibitor E64 (**6**) abolished its labeling by **15**, which shows that inactivation is dependent on the catalytic activity and labeling does not occur due to indiscriminate reaction with nucleophiles on the protein surface. In addition, ABP **15** has been shown to be capable of detecting cathepsin S specifically in a protein extract derived from human placental tissue.

The fluorophores employed in the ABPs **14** and **15** compromise their application for *in vivo* imaging, because their emission wavelengths are too short to ensure a sufficient light penetration from deeper tissues (James and Gambhir, 2012). Significant progress toward optical imaging of cysteine cathepsins has been achieved by using ABPs based on acyloxymethyl ketones. As this class of irreversible inhibitors interacts with the active-site thiol by nucleophilic substitution, they offer the opportunity to combine the fluorescence donor with a quencher that can be attached to the leaving group. Förster resonance energy transfer (FRET) in the unreacted probe will result in a strongly attenuated fluorescence of the unreacted probe, while the quencher leaves the enzyme-inhibitor complex upon inactivation which leads to enhanced luminescence of the donor. Consequently, target binding is coupled to signal amplification, which may account for good signal-to-noise ratios. Recently described probes of this type are compounds **16a** and **16b**. In **16a**, the commonly employed 2,6-dimethylbenzoyl moiety has been replaced by an electron-deficient 2,3,5,6-tetrafluorophenyl moiety in order to eliminate the potentially metabolically unstable ester linkage and to increase electronically and sterically the reactivity against the active-site cysteine (Verdoes et al., 2013). Both ABPs are equipped with a Cy5-derived fluorescence donor attached to the P1 position and Sulfo-QSY21 as quencher, which is tethered via an amide group and ethylene diamine or hexamethylene diamine linker to the *para* position of the tetrafluorophenyl or 2,6-dimethylbenzoyl group, respectively. The *in vitro* evaluation of **16a** was performed in RAW 264.7 cells, a mouse leukemic monocyte macrophage cell line, on the basis of in-gel fluorescence readouts in comparison to seven analogs that varied in the sulfonation of the QSY moiety, the chain length of the diamine linker and/or contained a 2,6-dimethylbenzoyl instead of the 2,3,5,6-tetrafluorophenyl moiety. It was observed that the presence of the sulfonic acid function at the QSY chromophore accounts for superior performance with regard to labeling of cysteine cathepsins in intact cells, while the change of the ethylene spacer to hexamethylene in the diamine linker impairs the probe's performance only to a minor extent. Furthermore, more hydrophobic analogs showed low level in-gel fluorescence signals that reached a maximum intensity at concentrations of 0.5–1.0 μM, which has been interpreted in terms of probe aggregation due to limited solubility. In contrast, **16a** showed brighter signals for the different cysteine cathepsins that increased up to a concentration of 5 μM. When the 2,3,5,6-tetrafluorophenyloxymethyl ketones were compared to their 2,6-dimethylbenzoyl-based counterparts it was obvious that the former type of probe led to a more uniform labeling of the cathepsins L, S, and B while the acyloxymethylketones reacted preferentially with cathepsin S and L. Surprisingly, the 2,3,5,6-tetrafluorophenyloxymethyl ketones were even capable of labeling cathepsin X, a papain-like cysteine protease which is generally difficult to target with inhibitors. It has been shown that concentrations of **16a** as low as 5 nM are sufficient to detect cathepsins L, S, B, and X in RAW 264.7 cell lysates. According to the authors, this phenomenon probably reflects the cellular localisation of the various cathepsins rather than differences in molecular recognition between the probe and the enzymes, as cathepsin X is supposed to reside on the cell surface. The investigation of the time course of cathepsin labeling by **16a** in live RAW 264.7 cells indicated that a rapid labeling of cathepsin X occurred, followed by cathepsins S, L, and B. Pretreatment of the RAW 264.7 cells with an excess of JPM-OEt (**7b**), a cell permeable “prodrug” of an irreversible inhibitor of the epoxysuccinyl chemotype, resulted in reduction of the in-gel fluorescence to 10% of the original signal. Preexposure of **16a** to serum for 4 h retained 80% of the cathepsin-targeting potential. In contrast, the identical treatment of the corresponding AOMK-based probe diminished the fluorescence signals associated with electrophoretic bands by 70%, which might due to hydrolytic cleavage of the ester linkage catalyzed by serum esterases. ABP **16a** was also investigated toward human monocyte-derived macrophages and a similar labeling profile was observed as in the murine cell line. Fluorescence microscopy studies with the human macrophages and **16a** led to red fluorescence in cell regions that have been identified as lysosomes. This signal was completely blocked in the presence of JPM-OEt (**7b**). The potential of **16a** for *in vivo* imaging of tumor-associated cysteine cathepsins was evaluated in a syngeneic orthotopic mouse model of breast cancer derived from murine 4T1 cells. Already 1 h *post injectionem* (p.i.) the margins of the tumor were visible with substantial contrast. Compound **16a** gives rise to a fluorescence signal in the tumor region whose intensity at 8 h p.i. was approximately 20-fold higher than that of its desulfo-hexamethylene-2,6-dimethylbenzoyl-based counterpart. This result was confirmed by *ex vivo* fluorescence measurements and reflects the *in vitro* performance of this probe. The *in vivo* targeting of cathepsins L, S, B, and X has been demonstrated by SDS-PAGE of the tumor homogenates. Interestingly, immunofluorescence staining for the macrophage marker CD68 in tissue sections from tumors pretreated with **16a** has revealed that labeling by the probe occurs almost exclusively in tumor-associated macrophages.

Furthermore, ABP **16a** has been evaluated in orthotopic mouse models of familial adenomatous polyposis and colitis-associated colorectal cancer (Segal et al., 2015). Upon intravenous injection of the probes, fluorescence accumulated in the malignant polyps but not in the surrounding benign intestinal tissue. The signal correlated well with the polyp diameter and was significantly lower under pre-treatment with compound **4**. For the colitis-related model of colon cancer the contrast between malignant and normal tissue was higher upon intra-rectal administration compared to intravenous injection. In this model of colon cancer, mainly cathepsin S was targeted by **16a**, as revealed by SDS-PAGE. Fluorescence microscopy on murine colon tissue sections indicated the colocalization of the probe with the immune cell marker CD45. Fluorescence micrographs obtained in immunohistochemical analyses of tissue sections derived from human colon polyps confirmed these results.

The motivation behind the design of compound **16b** was to achieve selective imaging of cathepsin S in the presence of other cysteine cathepsins. To this end, a fragment consisting of a 3-thenoyl cap and a *trans*-4-methylcyclohexyl residue to address the S^2^ pocket of cathepsin S have been employed in this compound (Oresic Bender et al., 2015). The peptide bond that connects the P^2^ and P^1^ moieties is replaced by a 1,4-disubstiuted triazole. This dipeptide-mimicking fragment has been identified to confer excellent selectivity to cathepsin S over the cathepsins B, K, and L (Patterson et al., 2006). Because the tetrafluorophenoxymethyl ketone warhead was found to be unfavorable regarding the selectivity toward cathepsin S, the 2,6-dimethylbenzoyl leaving group in combination with a hexamethylene diamine linker was employed. Incubation of RAW 264.7 cells with **16b** and subsequent analysis of cell lysates by SDS-PAGE confirmed its selectivity for cathepsin S over cathepsins B, L, and X. The probe was evaluated in the 4T1 murine breast cancer model. Tumor-associated fluorescence was clearly detectable 8 h p.i. but the intensity was 6 times reduced compared to **16a**, which reflects the selectivity of **16b** for cathepsin S. The probe's cathepsin S selectivity has been exploited for dual-color live cell cysteine cathepsin activity imaging in bone marrow-derived macrophages and dendritic cells. For this purpose, a pan-reactive green-emitting probe was prepared in which the Cy5 and Sulfo-QSY21 moieties of **16a** were replaced by BODIPY FL and BHQ-10, respectively. The immune cell lines were simultaneously incubated with this probe and **16b** at optimized concentrations for 2 h. Dual-color fluorescence microscopy revealed that cathepsin S colocalized with other cysteine cathepsins in the macrophage line while compartments that exclusively harbor cathepsin S seem to exist in dendritic cells (Oresic Bender et al., 2015).

#### Substrate-based probes

The concept of activatable fluorescence is easy to realize in substrates as hydrolysis of the peptide bond will lead to two cleavage products which will dissociate from each other. The advantages and disadvantages that are associated with the use of activity- or substrate-based probes for non-invasive optical imaging of the tumor-associated cysteine cathepsins have been exemplarily compared. It was concluded from this study that fluorescent ABPs exhibit more rapid and selective uptake into tumors as well as stronger signal contrast compared to substrate-based probes (Blum et al., 2009).

**Figure 7:**
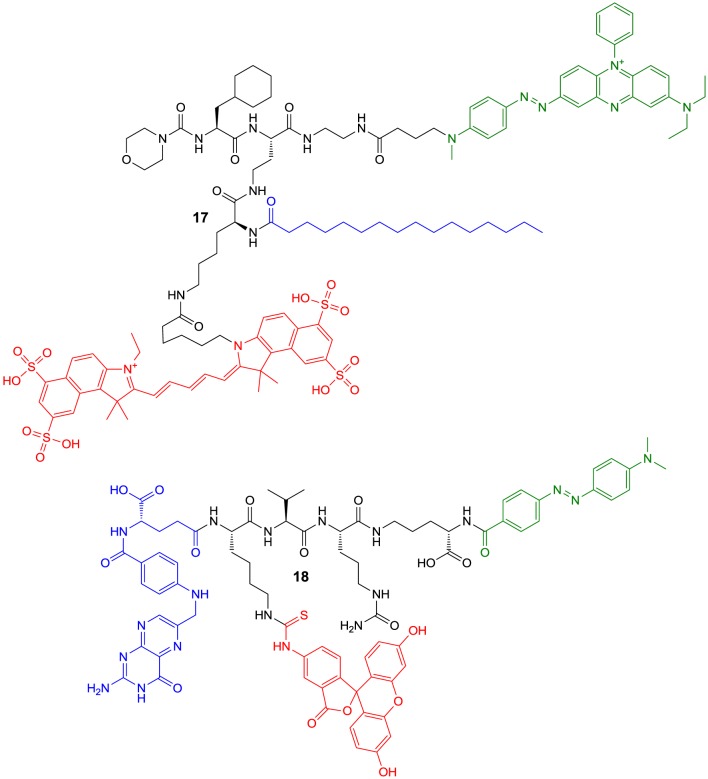
**Quenched substrate-based probes for optical imaging of cysteine cathepsins**. Emitting fluorophoric moieties are shown in red, chromophoric moieties that act as quencher are highlighted in green and additional targeting moieties are shown in blue.

However, the latter-type of imaging agents offer the potential advantage of enzyme-mediated signal amplification. A recently reported substrate-based probe is compound **17**, which has been developed for the imaging of tumor-associated cathepsin S (Figure 7) (Hu et al., 2014a). The compound has been designed on the basis of a potent cathepsin S inhibitor bearing cyclohexylalanine in P^2^ and a cyclic ketone in the P^1^ position. The ketone moiety gives rise to a reversible covalent interaction with the active-site cysteine by thiohemiketal formation. The N-terminal amino group of this dipeptide derivative is connected to a morpholine moiety via a urea linkage. A morpholino group in this position will be engaged in favorable interactions with the side-chain of Lys64 in the S3 region of cathepsin S via its oxygen atom (Pauly et al., 2003). Furthermore, cyclohexylalanine has been identified to be optimal to address the S^2^ pocket of cathepsin S (Ward et al., 2002). The combination of these two favorable moieties makes this inhibitor highly selective for cathepsin S (Link and Zipfel, 2006). In order to convert the inhibitor into a substrate and to allow the attachment of fluorophores and further targeting elements, the cyclic ketone in the P^1^ position was replaced by diaminobutyric acid. Such a strategy to design selective protease substrates starting from covalent inhibitors is termed reverse design. This concept seems to be promising because small peptide-derived inhibitors very often were structurally optimized using non-proteinogenic amino acids to achieve optimal targeting of protease subsites. It has been successfully applied for the design of optical imaging probes for cathepsin K and cathepsin S (Watzke et al., 2008). By employing lysine with orthogonally protected amino groups a Cy5.5 as NIR fluorophore and a palmitoyl group was attached to the diaminobutyric acid in the P^1^ position. Palmitoylation of the probe was done with the intention to facilitate its localisation to the cell surface, because the secreted cathepsin S will remain in proximity to the cellular membrane. Via an ethylenediamine linker the chromophore of black hole quencher 3 (BHQ-3) was attached as dark quencher to silence the Cy5.5 reporter. The kinetic characterisation of the substrate-based probe **17** toward hydrolysis catalyzed by the cathepsins B, K, L, S, and V has revealed that it is selectively cleaved by cathepsin S with a specificity constant *k*_cat_/*K*_m_ of 2700 M^−1^s^−1^. Interestingly, the cathepsin S-catalyzed hydrolysis of **17** was significantly enhanced in the presence of liposomes as membrane model. Microscopic evaluation of compound **17** together with its non-lipidated counterpart containing a maleimidylhexanoyl group instead of palmitoyl was done toward human THP-1 cell-derived macrophages. The studies indicated that both probes were cleaved rapidly by the cells and associated with them. Preincubation with cell-impermeable E64 (**6**) resulted in blocking of the cleavage of **17** only, whereas that of the non-lipidated probe was not influenced. In contrast, treatment with E64d (**8b**), a cell-permeable analog of E64 prevented the hydrolysis of both probes, which indicates that they are not recognized as substrates by other proteases. These results have been interpreted to indicate that the palmitoylated probe undergoes hydrolysis catalyzed by surface-located cathepsin S, whereas the non-lipidated probe is subjected to internalization before it is cleaved. Because compound **8b** inhibited the activation of both probes, they are probably not recognized as substrates by other proteases, and thus, cleaved by cathepsin S in a highly specific manner. Performing the experiment at 4°C did not lead to activation of the non-lipidated probe, whereas the signal from activation of **17** was restricted to the cell membrane due to attenuated endocytosis. *In vivo* evaluation in 4T1 tumor-carrying mice revealed that for both probes a significant fluorescence signal was observable in the tumor 30 min p.i. that did not vanish up to 24 h p.i. At his time the signal caused by **17** was as twice as high compared to its non-lipidated counterpart. The tumor-muscle ratio of **17** reached a maximum of 18 after 5 d p.i. The non-lipidated probe was also detectable in the kidneys, whereas the uptake in other organs was very low. Compound **17** is taken up by the kidneys to a similar extent as its non-lipidated counterpart, whereas lipidation resulted in a significantly higher uptake in all other investigated organs (lung, liver, spleen), but the levels were greatest for the tumor. The significantly increased tumor uptake of **17** compared to the non-lipidated probe was confirmed in *ex vivo* investigations of tumor sections by fluorescence microscopy (Hu et al., 2014a).

A probe of similar composition as **17** is compound **18**, which has been described by Tian et al. (2014). The similarity is due to the fact that **18** contains a pteroyl group as targeting unit additional to the cathepsin cleavage site, in analogy to the palmitoyl residue of **17**. In difference, probe **18** has been designed to primarily target the folate receptor by the pteroylglutamyl group. The cellular uptake of folic acid and its derivatives is mediated by two transport systems, the reduced-folate carrier (SLC19A1) and the glycosyl phosphatidylinositol (GPI)-anchored membrane folate receptors α (FOLR1) and β (FOLR2). While the former is expressed ubiquitously and facilitates bidirectional diffusion of folates across the plasma membrane, the latter affect the unidirectional uptake of folic acid by receptor-mediated endocytosis via the endosomal recycling pathway (Shen et al., 1997; Matherly et al., 2007). Folate receptor expression in homeostasis is restricted to the lung, kidneys and placenta. An overexpression of the folate receptor can occur in neoplastic tissue, especially epithelial cancers such as ovarian, colorectal, and pancreatic carcinoma (Assaraf et al., 2014).

The design of probe **18** aims at coupling the targeting of folate receptor to activation by intracellular cathepsin B upon receptor-mediated internalization. Compound **18** is constituted by the peptidic cathepsin-responsive core moiety harboring valine and citrulline to target the S^2^ and S^1^ subsites of cathepsin B, respectively. To target the primed binding regions, ornithine is employed, which is connected to citrulline via its δ-amino group. Notably, ornithine can be considered as dipeptide mimetic in this context, as its carboxy group might be able to target the histidine residues in the occluding loop. Equipped with fluoresceine conjugated to a lysine side chain, the probe is quenched in the uncleaved state by Förster resonance energy transfer to a dabcyl group, which is attached at the α-amino group of ornithine. The folate group has been linked to the N-terminus via the side chain of its glutamate moiety. Exposure of **18** to cathepsin B (400 nM) resulted in a 10-fold increase in fluorescence due to dequenching upon cleavage within 250 min. The compound has been characterized to be stable in blood plasma for at least 24 h, which favors its *in vivo* application. The cell uptake of **18** has been studied with KB cells, a human epidermoid carcinoma cell line expressing the folate receptor, using FACS analysis. The uptake has been confirmed to be concentration-dependent and the cellular fluorescence was much lower when the folate group of **18** was replaced by an acetyl residue. Furthermore, preincubation of the KB cells with 50 μM folic acid reduced the fluorescence by approximately 70%. In accordance with this result, the uptake of **18** was clearly reduced when cells expressing low levels of the folate receptor such as MCF-7 breast cancer cells and mouse embryonic fibroblasts were used instead of KB cells. The intracellular activation of the probe was demonstrated by repeating the FACS analysis at 0°C together with the analog lacking the dabcyl group. The fluorescence intensity of the cells incubated with **18** was articulately reduced over the ones treated with its unquenched counterpart, because the ATP-consuming internalization of the probe-folate receptor complex is attenuated at this temperature. The intracellular activation of **18** was furthermore confirmed by microscopic studies in KB cells, as after 30 min green fluorescence at the cell surface was only observable for the unquenched counterpart and not for compound **18**. The microscopic investigations also revealed an unspecific binding for the folate lacking analogs of **18** at higher concentrations (200 nM). This finding has been attributed to the hydrophobic character of the fluoresceine and dabcyl groups and indicates a general drawback of optical imaging probes, because the bulkiness and hydrophobicity of the required chromophores may negatively influence their behavior in biosystems (Tian et al., 2014).

**Figure 8:**
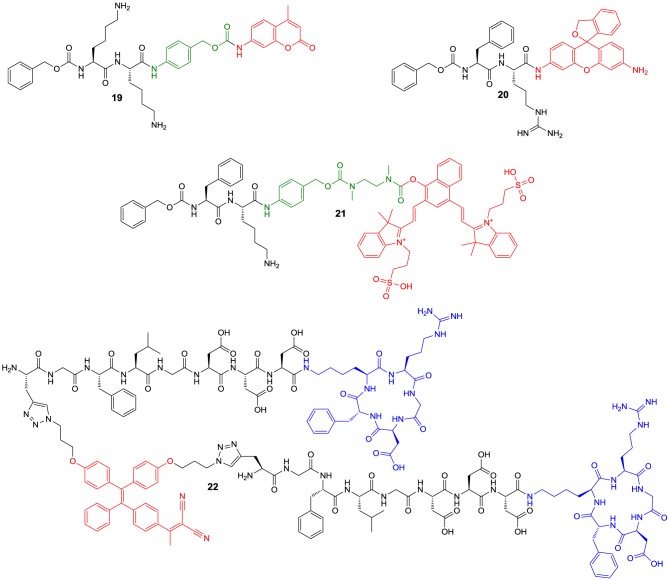
**Fluorogenic substrate-based probes for targeting of cysteine cathepsins containing only one luminophor**. Emitting fluorophoric moieties are shown in red, moieties that act as spacer are highlighted in green and additional targeting moieties are shown in blue.

Despite quenched substrates offer the advantage of enzyme-catalyzed signal amplification, substrate-based imaging is also possible with probes that contain a fluorogenic leaving group in the absence of a quencher. This principle is based on the deactivation of substituents that exert a +M effect on the aromatic core of fluorophoric entities by the attachment of acyl groups to these substituents. Cleavage of those acyl groups by the action of enzymes will result in unblocked electron donation to the fluorophore and thus increased fluorescence. This principle has been used extensively for the activity determination of proteases and other enzymes that catalyze acyl transfer reactions (Grimm et al., 2013) while reports concerning their application for imaging purposes are scarce. Probes of this type that have been recently developed for cellular imaging are compounds **19** and **20** (Figure 8). Development of compound **19** started from the established dipeptidic fluorogenic substrate Z-Arg-Arg-AMC, which is sensitive and selective for the fluorimetric detection of cathepsin B activity. For the sake of better synthetic accessibility, its two arginine residues were replaced by lysines. This structural change had almost no influence on the *k*_cat_/*K*_m_-values (148 and 167 mM^−1^s^−1^ for Z-Lys-Lys-AMC and Z-Arg-Arg-AMC, respectively). To increase the stability toward degradation by other proteases and the affinity toward cathepsin B a *para*-aminobenzyloxycarbonyl (PABA) linker was placed between the peptidic and AMC moiety, as this will enable a more effective targeting of the primed binding sites (presumably S^1′^ and S^2′^) of cathepsin B (Chowdhury et al., 2014). PABA is a self-destructive linker, as release of its amino group will result in spontaneous fragmentation due to imino-quinone methide formation, which has been successfully used for prodrug design (Carl et al., 1981). Indeed, **19** showed a slightly increased *k*_cat_/*K*_m_-value (231 mM^−1^s^−1^) than Z-Lys-Lys-AMC, which seems to be due to a decrease in *K*_m_. Noteworthy, the introduction of the PABA linker was not tolerated by cathepsin L as the analog of **19** containing Phe instead of Lys in P^2^ (Z-Phe-Lys-PABA-AMC) was not converted by this enzyme. Therefore, the combination of the two Lys residues with the self-destructive PABA linker makes **19** to a substrate which is cleaved by cathepsin B with excellent selectivity over cathepsins L and S. Probe **19** was also effectively cleaved by lysates of HeLa cervical tumor cells. Pretreatment of HeLa cells by the cell-permeable pro-inhibitors E64d (**8b**) or CA074Me (**9b**), which inhibit several cysteine cathepsins or selectively cathepsin B, respectively, after intracellular activation by esterases, resulted in a complete blocking of the conversion of **19**. Fluorescence microscopic observation of live HeLa cells in the presence of Z-Arg-Arg-AMC or Z-Phe-Arg-AMC did not result in the detection of any cellular fluorescence. Contrary to this, the three-component probe **19** containing the PABA linker led to blue fluorescent cells, while no intracellular fluorescence could be visualized in the presence of CA074Me (**9b**) or E64d (**8b**). Similar results were obtained when the HeLa cells were replaced by the Her2-positive breast cancer cell line MDA-MB-231-H2N. These results clearly indicate that **19** undergoes cathepsin B-dependent activation inside the cells and that the PABA linker obviously facilitates membrane permeation of the peptidic probe. When Z-Phe-Lys-PABA-AMC was investigated in the same manner, it was revealed that its cellular activation was only completely blocked by treatment with E64d, while the presence of CA074Me resulted in considerable residual cellular fluorescence. These observations suggest that the latter probe is activated by cathepsin B and other cysteine cathepsins, which is in accordance to results from kinetic investigations and cell lysate experiments. Furthermore, Z-Phe-Lys-PABA-AMC compromises cell viability in both cell lines as assessed by the MTT assay, while **19** and the ordinary two-component probe Z-Arg-Arg-AMC are well-tolerated (Chowdhury et al., 2014).

While 7-amino-4-methylcoumarin is an advantageous fluorophore for protease activatable probes due to its small size, its blue fluorescence and rather short excitation wavelength are adverse properties. Alternatives can be found by rhodamine derivatives, which exhibit two amino groups attached to the xanthenyl system and offer the opportunity of longer wavelength excitation/emission together with high quantum yields for fluorescence. Peptidic moieties can be attached to the two amino groups of rhodamines which will result in fluorescence attenuation and illumination upon protease-mediated cleavage (Leytus et al., 1983; Rothe et al., 1992). However, a drawback of such probes is that consecutive removal of two peptide residues will result in mono-acylated and completely deacylated rhodamine species which will differ in their fluorescence properties. To tackle that problem, Fujii et al. (2014) developed activatable probes based on “reduced” rhodamine green (rhodamine green is also known as rhodamine 110), in which the carboxy group is replaced by a hydroxymethyl residue. Typical for xanthene dyes, this hydroxymethyl rhodamine green (HMRG) exists in solution in a ring-chain tautomeric equilibrium between a non-fluorescent spirocyclic form and a highly fluorescent quinoid open form. At physiological pH, the equilibrium lies on the side of the open form, which is characterized by favorable photophysical properties such as high fluorescence quantum yield (Φ_fl_ = 0.81) and long fluorescence lifetime (τ = 3.8 ns) (Sakabe et al., 2013). Mono-acylation of one of the amino groups will stabilize the non-fluorescent spirocyclic form and thus result in strong attenuation of fluorescence. Fujii et al. aimed to exploit this principle for the design of cathepsin B-activatable probes in the context of optical tumor imaging. To this end, they attached the Z-Phe-Arg (as realized in compound **20**) and Z-Arg-Arg moiety to HMRG. The extinction coefficient of free HMRG is more than 400 times higher than that of **20** (at λ_max_ = 501 and 497 nm, respectively). The kinetic evaluation toward the cathepsins L, B, S and K was done at pH 5.5 and pH 7.4, to simulate intralysosomal and cytosolic/extracellular pH-values, at a single substrate concentration of 3 μM. Cathepsin B rapidly converted **20** at both pH-values, whereas the cleavage of Z-Arg-Arg-HMRG was only effective at pH 5.5. At this pH conversion of **20** by cathepsin L was slower than that by B, whereas Z-Arg-Arg-HMRG was cleaved with much lower velocity. The cleavage of **20** catalyzed by cathepsin L at pH 7.4 was very slow, probably due to the fact that cathepsin L undergoes irreversible inactivation at pH-values greater than 6.5 (Brömme et al., 1993). Compound **20** was also converted by cathepsin K at both pH-values but was no effective substrate of cathepsin S. Incubation of **20** with SK-OV-3 human ovarian tumor cells resulted in microscopically visible cell-associated fluorescence, which was attenuable by treatment with the broad-spectrum cell-permeable fluoromethylketone inhibitior Z-Phe-Phe-FMK (**1a**). It was concluded that cleavage of **20** occurs intracellularly, because the treatment with membrane-impermeable inhibitor CA074 (**9a**) was of negligible influence. Intraperitoneal injection of **20** into mice bearing peritoneally implanted SK-OV-3 tumors, a model for human ovarian cancer, resulted in a clear visualization of the tumor nodules with a tumor-to-background ratio of 4.2 The stability of **20** in peritoneal liquid has been confirmed independently and the green HMRG fluorescence colocalized with the tumor nodules when SK-OV-3 cells transformed with red fluorescent protein (RFP) were implanted.

The disadvantages of the discussed non-quenched cathepsin-responsive probes are due to the fact that the emission wavelengths of their fluorophores are too short for *in vivo* imaging. Fluorophores with emission wavelengths in the near infrared (NIR) range can be found within the class of cyanine dyes, which are employed in the FRET-based probes **16** and **17** discussed above. Despite these fluorophores are characterized by high extinction coefficients, acylation-based attenuation of fluorescence is difficult to realize, in contrast to luminescent groups on the basis of aromatic amines such as AMC and HMRG. To overcome this drawback, Kisin-Finfer and Shabat (2013) set out to develop fluorophores where three consecutive carbons of the polymethine chain of cyanine dyes are part of a napthalene ring. To this end, they reacted 4-hydroxynaphthalene-1,3-dicarbaldehyde with two equivalents of 3-(2,3,3-trimethyl-3H-indol-1-ium-1-yl)propane-1-sulfonate in a Knoevenagel-type condensation. The obtained product is a representative of the so called “donor-two-acceptor” NIR dyes, referring to the electron-donating hydroxyl group and the two indolium acceptor moieties (Karton-Lifshin et al., 2012), and shows a pH-dependent fluorescence with an emission wavelength of 740 nm and a large Stokes shift of 80 nm. Notably, only the deprotonated phenolate form is fluorescent. In consequence, acylation of the OH group will lead to strongly attenuated fluorescence, which can be exploited to design hydrolase-sensitive probes as it has been realized in compound **21** for imaging of cathepsin B (Kisin-Finfer et al., 2014). Considering the steric demand of the fluorophore, its conjugation to the cathepsin-responsive Z-Phe-Lys moiety was done via a self-immolative linker which resembles in part the PABA linker contained in **19** (Chowdhury et al., 2014). In difference, a *N*,*N*'-dimethylethylenediamine-carbonyl spacer follows the PABA element (Shabat et al., 2004). Analogously to **19**, cathepsin B-mediated release of the aromatic amine will result in the formation of imino quinone methide and carbon dioxide. This reaction step triggers in turn the release of the phenolic hydroxyl group via cyclisation by attack of the secondary amine nitrogen to the carbamate carbon under concomitant formation of a five-membered cyclic urea. Apart from creating space between the fluorophore and the cleavage site, this strategy ensures that the two functional entities are joined by sufficiently stabile amide and carbamate linkages instead of a labile aryl ester bond. Based on visual inspection, incubation of **21** with rather high concentrations of cathepsin B led to a strong red fluorescent solution after 4 h upon excitation at 661 nm, while no background luminescence was observable in the absence of the enzyme. This result is in contrast to the analogous quenched (FRET-based) probe, which has been designed to contain a Cy5 fluorophore in place of the Z group and a heptamethine-cyanine dye as quencher attached via the PABA linker. In consequence, the *in vivo* performance of **21** upon intratumoral injection into 4T1 tumor-bearing mice was more favorable over the corresponding quenched probe as it exhibited a considerably enhanced signal-to-noise ratio.

A further type of fluorescent probe whose optical properties can be triggered upon cathepsin-catalyzed cleavage in the absence of a quencher is compound **22** (Yuan et al., 2015). Of note, the enhancement in fluorescence in this probe is not mediated by electronic changes due to bond cleavage, but by a change in solubility. Aggregation of fluorophores usually leads to attenuation in the fluorescence intensity, a phenomenon which is called concentration quenching (Chaudhuri, 1959). Under certain circumstances, the contrary phenomenon of aggregation-induced emission (AIE) can occur (Ding et al., 2013; Mei et al., 2014). This can be observed for fluorophores based on the tetraphenylethylene scaffold. Free in solution, the quantum yield is low because the energy of the excited state is dissipated into the rotation of the phenyl groups. In the aggregated state these rotations are restricted due to intermolecular interactions and thus the fluorescence quantum yield increases. To exploit this principle for tumor imaging, the tetraphenylethylene scaffold has been functionalized with a recognition sequence for cathepsin B-catalyzed cleavage. This has been achieved by attaching azidopropyl groups to the luminophoric core. The required dihydroxy derivative has been accessed by a crossed McMurry reaction from the corresponding benzophenone building blocks. The fluorogenic core has been conjugated to a peptidic construct consisting of the GF↓LG tetrapeptide unit, which serves as the recognition site for cleavage by cathepsin B (Rejmanova et al., 1983). The N-terminal propargylglycine allows conjugation to the azido-functionalized fluorophore by azide-alkine 3+2 cycloaddition. Via a C-terminal linker consisting of three aspartic residues, which confer hydrophilicity to the probe, the cyclopentapeptide c[fKRGD] is attached via its lysine side chain. The latter unit is intended to target the α_*v*_β_3_ integrin type receptor, which is overexpressed in several tumor cells. Thus, compound **22** represents a dual-targeted probe similar to the folate conjugate **18** with the cyclic RGD unit corresponding functionally to the folate moiety. The azido-functionalized tetraphenylethylene derivative has been characterized regarding their media-dependent luminescence and has been found to be almost non-fluorescent dissolved in DMSO. Upon addition of water, the fluorescence steadily increased and at a mole fraction of 99% water the emission intensity was 105-fold higher than in pure DMSO. The emission spectra are characterized by maxima at 615 nm upon excitation at 365 nm. Notably, the emission is independent of pH in the range of 7.4 to 5.0. Due to its hydrophilicity, probe **22** is virtually non-fluorescent in aqueous solution in the uncleaved state. Incubation of **22** (5 μM) with cathepsin B (1 μg/mL) for 60 min at a pH 5.0 and 37°C resulted in a 35-fold increase of fluorescence. The performance constant of **22** toward cathepsin B has been determined to be 142 M^−1^s^−1^. RP-HPLC analysis of cathepsin B-incubated **22** indicated elution at considerably higher retention times and thus higher hydrophobicity than in the uncleaved state. Hydrophobic clustering upon cleavage has been confirmed by laser light scattering and atomic force microscopy investigations. Activation of **22** can also be achieved by lysates derived from the human mammary carcinoma cell lines MDA-MB-231 and MCF-7, while the increase in fluorescence with lysates of human embryonic kidney cells 293T and tumor cells that have been preincubated with CA074Me (**9b**) is considerably less strong. Fluorescence microscopic analysis indicated that cell uptake of **22** occurs to a substantial extent only in the α_*v*_β_3_ positive MDA-MB-231 cells. Cell-associated red fluorescence in these cells declines upon preincubation with either or both free RGD cyclopentapeptide and compound **9b**, which has been confirmed quantitatively by FACS analysis. Fluorescent molecules can potentially act as photosensitizers for photodynamic therapy because transition from the excited triplet state to the ground state can be coupled to conversion of triplet oxygen into highly reactive singlet oxygen. To evaluate the probe's potential toward this application, Yuan et al. monitored the release of reactive oxygen species in the presence of **22** and cathepsin B with 1,3-diphenylisobenzofuran (DBPF) as indicator. The cleaved probe exhibited a photosensitizing activity which was abolished in the presence of ascorbic acid. Investigation of the light-mediated cytotoxicity of **22** toward MDA-MB-231 cells has revealed that under irradiation a concentration of 5 μM can reduce cell viability by more than 50%, while in the dark the toxic effects are minimal. The phototoxicity has been demonstrated to be receptor-mediated, as the α_*v*_β_3_ negative MCF-7 and 293T cells are viable in the presence of **22** under irradiation at 365 nm. Based on these *in vitro* results, **22** can be considered as a potential theranostic agent, because it combines functional imaging with targeted photodynamic ablation. To exert photodynamic effects *in vivo*, excitation wavelengths above 600 nm are desirable, which limits the applicability of **22** for therapeutic purposes.

**Figure 9:**
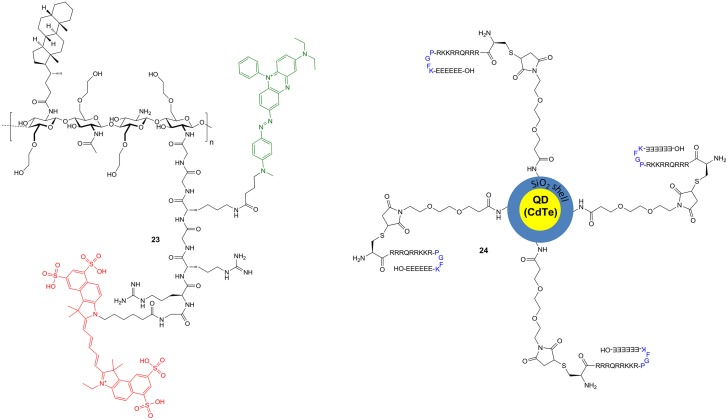
**Polymeric/particulate fluorescent probes for cathepsin B**. Due to its complexity and size, probe **24** is only schematically represented.

The development of imaging probes is not restricted to compounds of low molecular weight and a few polymeric and particulate probes for the *in vivo* detection of cathepsin B have been described. Among the first macromolecular probes for this purpose is a poly-L-lysine-based polymer, whose amino groups were partially functionalized with Cy5.5. The spatial proximity of the fluorophores led to internal fluorescence quenching and unmodified lysines served as recognition sites for cathepsin B-catalyzed cleavage, resulting in enhanced fluorescence. *In vivo*, probe activation within a mouse model of human breast cancer correlated with the tumor-associated cathepsin B activity (Bremer et al., 2002). In a different approach, a nanoparticle consisting of *O*^6^-hydroxyethylchitosane (chitosane = deacetylchitin) has been functionalized with the heptapeptidic cathepsin B recognition sequence GRR↓GKGG to a substitution degree of 21 peptide chains per 1 molecule of chitosane-based nanoparticle (Figure 9, compound **23**) (Ryu et al., 2011). The particles exhibited a molecular weight of 345 kDa and a spherical shape with a diameter of 280 nm. The hexapeptide was equipped with Cy5.5 at the N-terminus and BHQ-3 at the side chain of Lys as fluorescence emitter and quencher, respectively. Exposure of the nanoprobe to 1.5 nM of cathepsin B resulted in an approximately 15-fold increase in fluorescence, while no significant dequenching was observed with cathepsin L, the aspartic protease cathepsin D or cathepsin B in the presence of Z-Phe-Lys-FMK (**1b**) as inhibitor. The uptake of the cathepsin B-responsive nanoparticle in SCC7 murine squamous cell carcinoma was accompanied by increasing red fluorescence, which colocalized with the green fluorescence for immunohistochemical detection of cellular cathepsin B. The cell-associated Cy5.5 fluorescence signal could be blocked by treatment with **1b** and the particulate probe resulted in stronger NIR fluorescence than the isolated, soluble quenched peptide. The *in vivo* evaluation of the nanoprobe **23** was performed in mice bearing SCC7 tumors. The tumor-associated fluorescence increased up to 6 h p.i. Regarding the influence of inhibitor treatment and the comparison with the non-particulate probe the results obtained *in cellulo* could be confirmed. The fluorescence in non-target organs was more than twice as low as in the tumor. In a further study, this probe was re-evaluated in mice bearing tumors derived from HT29 human colorectal carcinoma cells (Ryu et al., 2014). Intravenous injection of the nanoprobe into these animals resulted in NIR fluorescence signals that were 17-fold higher than in the control animal where the tumor-associated cathepsin B activity has been blocked by intratumoral injection of **1b**. Furthermore, the probe's capability to image the metastasis-associated cathepsin B activity in three different mouse models of metastatic tumors was evaluated. The first model concerned metastasis to the liver of 4T1-luc2 cells injected into spleen. Liver metastases were detectable 10–14 days after tumor cell injection into the spleen by luciferase-based bioluminescence imaging. Systemic administration of the cathepsin B-responsive nanoparticle resulted in a NIR fluorescence that matched the bioluminescence signal, while only minimal fluorescence was detectable upon injecting the probe into normal mice. The increased expression of cathepsin B in liver metastases has been confirmed by Western blot analysis. Similar results were obtained when murine red-fluorescent-protein-expressing RFP-B16F10 melanoma cells or highly malignant HT1080 cells were injected into the tail vein and into the peritoneum, respectively. The former experiment models the process of metastasis to the lung and the latter that of peritoneal metastasis. The uptake of the cathepsin B-responsive nanoprobe has been investigated in all these four tumor cell lines by fluorescence microscopy. A strong cell-associated Cy5.5-derived fluorescence was observable for the tumor cells, whereas only faint signals were detectable for the cathepsin B-negative NIH3T3 mouse embryo fibroblasts.

The design of cathepsin-sensitive nanoprobes not only offers the opportunity of functional tumor imaging, but also the selective delivery of chemotherapeutic agents into the tumor. The creation of such a cathepsin-responsive particulate nanocarrier was the motivation behind the work of Li et al. (2014). The nanoparticle has been composed of a quantum dot (QD) core structure based on cadmium telluride (CdTe). Compared to classical organic fluorophores, QD's exhibit broad absorption and narrow emission spectra and enhanced brightness. Their emission maxima can be tuned between 450 and 850 nm by changing their size (Lakowicz, 2010; Barreto et al., 2011; Wegner and Hildebrandt, 2015). The CdTe core was coated with mesoporous SiO_2_. The mesoporous silica shell bears the potential to adopt doxorubicin, a member of the anthracycline class of DNA-targeting antitumor antibiotics, which is intended to be delivered to the site of action in the cell nucleus with the help of the nanocarrier. A maleimido group was conjugated to the amino-functionalized surface of the silica shell via a 4,7-dioxanonanoyl linker. The maleimido group served as an adapter for the attachment of the 20 mer peptide H-CRRRQRRKKR-PGFK↓-EEEEEE-OH via Michael addition of its N-terminal cysteine residue (Figure 9, compound **24**). The peptide chain consists of an N-terminal cell-penetrating sequence which is attenuated by the C-terminal polyanionic hexaglutamate chain. Both elements are linked by the PGFK tetrapeptide which serves as recognition site for cathepsin B-mediated cleavage. Scanning and transmission electron microscopy indicated a confined size distribution of the nanoparticles with an overall diameter of 40 nm, which was considered as a suitable size to achieve effective cell penetration and targeted nuclear delivery. The fluorescence of the particle due to the CdTe QD core is around 560 nm. The empty nanocarrier was evaluated using confocal laser scanning microscopy toward human A549 adenocarcinoma cells as cathepsin B positive cell line and NIH3T3 mouse embryo fibroblasts as negative control. After 24 h of incubation, the yellow QD fluorescence is mainly located inside the nucleus of A549 cells, whereas in the cathepsin B negative NIH3T3 cells the fluorescence is restricted to the cytoplasm. A similar distribution of the nanoparticle can be observed in A549 in the presence of the broad-spectrum cysteine cathepsin inhibitor antipain (**12**). These results suggest a cathepsin B-mediated direction to the cell nucleus for the nanoparticle. Loading of doxorubicin to the nanocarrier can be achieved to a degree of 10.7%. Release of doxorubicin from the nanocontainer is clearly accelerated at pH 5.5 in the presence of cathepsin B.

In accordance with the above results, after 24 h of incubation with the loaded nanocarrier, red doxorubicin fluorescence in the nucleus was only detectable in the A549 cells and not in the NIH3T3 cells. In contrast, upon incubation with free doxorubicin over the same time, red fluorescence was detectable in the nuclei of both cell lines. Exposure of the tumor cell lines A549 and A2780 to the doxorubicin-loaded particles at a concentration of 10 μg/mL led to compromised cell viabilities that were lower than under equal concentrations of free doxorubicin. This difference was especially noticeable for the doxorubicin-resistant cell line A2780/Adr, where free doxorubicin elicits only minimal effects, due to the mechanisms of multidrug resistance, which actively transport chemotherapeutic agents out of the tumor cell. For comparison, the empty nanocarrier displays only minimal toxicity toward the investigated cell lines. These results allow concluding that the enzyme-responsive nanocarrier has the potential to considerably improve chemotherapy by selectively delivering cytostatic drugs to the nucleus of cathepsin B positive tumor cells and circumventing mechanisms of drug resistance.

### Radiotracers

Compared to fluorescence-based methods, detection on the basis of ionizing radiation emitted by nuclides that undergo radioactive transformations is generally more sensitive. Therefore, probes consisting of molecules labeled with radionuclides, so called radiotracers, offer the possibility of sensitive detection both for *in vitro* investigations and imaging applications with very often minimal structural changes compared to the unlabeled ligand. The scope of application for radiotracers is determined by the nuclear-physical properties of the radionuclides they are labeled with. Table 1 summarizes the properties of radionuclides that have been employed for the design of cysteine cathepsin-targeting radiotracers. Long-lived radionuclides that exhibit low particle energy such as tritium and carbon-14 offer the possibility of sensitive detection of enzyme inhibitor complexes and provide the possibility to study the biodistribution and biotransformation of cathepsin inhibitors of potential therapeutic interest by radio-HPLC and *ex vivo* autoradiography (Solon, 2012; Uhl et al., 2015). Their use for imaging is not possible because the β^−^ particles are difficult to measure outside the organism. For this purpose, short-lived radionuclides that undergo transformations accompanied by the emission of γ-ray photons can be applied for single-photon computed tomography (SPECT). The other modality of nuclear imaging, positron emission tomography (PET), depends on the use of tracers labeled with neutron-deficient radionuclides that stabilize by the transformation of a proton into a neutron under the emission of a positron. Because positrons are the anti-particles to electrons, the emitted positron will undergo annihilation upon collision with an electron under the emission of two γ-ray photons of 511 keV in the angle of nearly 180° to each other. This physical phenomenon allows the construction of images and time-activity curves by coincidence detection of the two photons using a circular array of scintillation detectors (James and Gambhir, 2012). This makes PET to a unique method for quantitative tracing compounds in living organisms (Bergmann and Pietzsch, 2005). Of note, far reaching progress in clinical PET technology has also led to the design and development of dedicated small animal PET systems that now are abundantly prevalent in biomedical and pharmaceutical research. This enables researchers to perform preclinical PET studies on pharmacokinetics of novel radiotracers with high spatial and temporal resolution, in living subjects such as in rodent laboratory models (Brust et al., 2014).

**Table 1:** **Properties of radionuclides used in cysteine cathepsin-targeting radiotracers**.

**Radio-nuclide**	***T*_1/2_**	Decay mode (abundance)^*^	Particle/radiation energy (*E*_mean_ in keV)^**^	**Production (nuclear reaction)**	**Modality**	**Labeling chemistry**
^3^H	12.32 a	β^−^	5.7	Reactor (^6^Li(n, α))	*In vitro*	Covalent
^11^C	20.4 min	β^+^ (99.8%)	386	Cyclotron (^14^N(p, α))	PET	Covalent, mostly electrophilic
^14^C	5730 a	β^−^	49.5	Reactor (^14^N(n, p))	*In vitro*	Covalent
^18^F	109.8 min	β^+^ (97%)	249	Cyclotron (mostly ^18^O(p, n))	PET	Covalent, mostly nucleophilic
^64^Cu	12.7 h	β^+^ (18%)	278	Cyclotron (^64^Zn(p, n))	PET	Coordinative
^125^I	59.4 d	EC	26.4	Reactor (^124^Xe(n, γ)^125^Xe(EC))	*In vitro* (SPECT)	Covalent, mostly electrophilic
^177^Lu	6.7 d	β^−^, γ	133 (β^−^), 208 (γ)	Reactor (mostly ^176^Lu(n, γ))	SPECT, therapy	Coordinative

* *For clarity, only those nuclear transformations that are relevant for the imaging process are shown*.

** *Data have been retrieved from the Brookhaven National Nuclear Data Center^6^*.

Furthermore, the short half-lives of the most employed PET nuclides imply that the administered amounts of labeled substances are usually far below target saturation so that a biochemical influence of the studied process does not occur (McCarthy et al., 1994). Despite higher sensitivity of radiotracer-based imaging, luminescence-based imaging offers the advantage of higher spatial resolution. To take advantage of both imaging modalities, fluorophores and radiolabels can be combined in one molecule. Even exclusively radiolabeled compounds can be used for OI, because the movement of high-energetic charged particles through matter is accompanied by the emission of UV and visible light known as Čerenkov radiation. The Čerenkov photons can be detected by a CCD camera, provided that the energy of the charged particle emitted upon radioactive transformation is greater than 219 keV (Thorek et al., 2012). Therefore, optical and nuclear imaging modalities are considered complementary to each other (James and Gambhir, 2012; Chin et al., 2013; Nordstrom et al., 2013; Seibold et al., 2014).

#### ^125^I-labeled compounds

**Figure 10:**
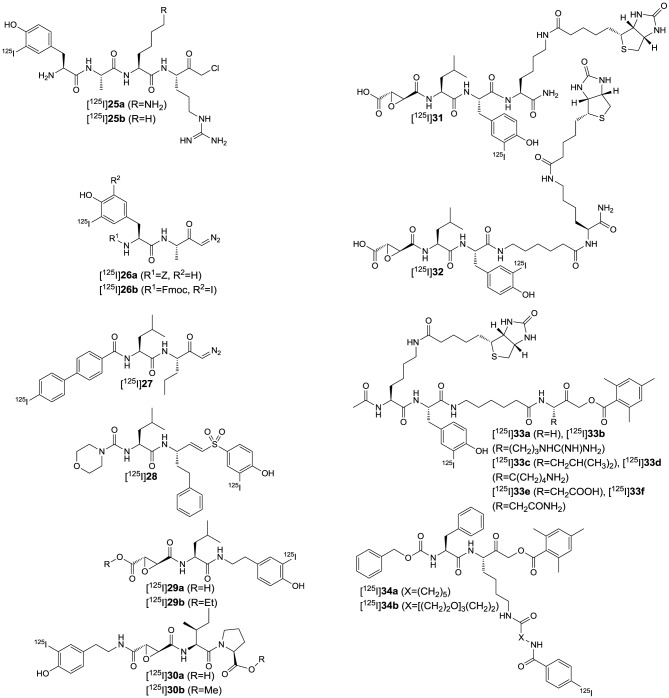
**Cysteine-cathepsin targeting radiotracers labeled with I-125**.

The development of radiotracers which target cysteine cathepsins started three decades ago with a tetrapeptide-derived chloromethylketone-based inhibitor labeled with iodine-125 ([^125^I]**25a**, Figure 10). Iodine-125 is one of four biomedically relevant iodine radioisotopes (Wilbur, 1992; Mier and Eisenhut, 2011). It stabilizes by electron capture, where a proton combines with an electron of the inner shells, usually the K-shell. The formed electron gap is filled under the emission of X-ray photons. While iodine-125-based radiotracers are typically employed for *in vitro* investigations, this radionuclide also offers the possibility of small-animal SPECT imaging. Radioiodination of biologically relevant compounds is very often achieved by reacting a hydroxyphenyl moiety with an iodine−+1 species which is generated from radioactive iodide and a mild oxidizing agent such as iodogen. This principle has been used to introduce iodine-125 into compounds [^125^I]**25**, [^125^I]**26** and [^125^I]**28**–**33** (Figure 10), which are all irreversible inhibitors that covalently target different cysteine proteases. Compounds [^125^I]**25a** and **b** seem to be the first described radiotracers to target cysteine cathepsins (Docherty et al., 1983, 1984). They have been used to identify a protease of 31.5 kDa of the granule fraction of rat Langerhans islets as cathepsin B by immunoprecipatation and SDS-PAGE. It is known that peptide-derived chloromethylketones can also interact covalently with the active site of serine proteases and Docherty et al. (1983) demonstrated the reactivity of [^125^I]**25a** toward trypsin. Therefore, doubts in the selectivity and the high intrinsic reactivity of this radiotracer motivated Mason et al. (1989b) to prepare [^125^I]**26a** as a ^125^I-labeled diazoketone. The specific activity of [^125^I]**26a** was determined to be 222 MBq/μmol. Deiodo-**26a** has been shown to be an effective inactivator of cathepsins L and B with selectivity over calpain, a cytosolic Ca^2+^-dependent cysteine protease. Iodination to non-radioactive **26a** even improves the inhibitory properties (Crawford et al., 1988). The radiotracer [^125^I]**26a** has been used to identify cathepsin L and B in Kirsten-virus-transformed NIH3T3 cells by SDS-PAGE. Electrophoretic separation of trichloroacetic acid precipitates derived from extracts of cells that were incubated with [^125^I]**26a** indicated the presence of one active cathepsin B species of 33–35 kDa and two active forms of cathepsin L of 30 and 23 kDa. For comparison, immunoprecipitates of labeled proteins obtained by pulse-chase incubation with [^35^S]methionine were separated by SDS-PAGE. This resulted in the detection of a 39 and a 36 kDa species which have been assigned to the intracellular precursors of cathepsin B and L, respectively. Radiotracer [^125^I]**26a** was furthermore applied to investigate the presence of active cathepsins B and L in homogenates of human *post mortem* tissues of heart and skeletal muscle, brain, kidney, pancreas, and spleen (Mason et al., 1989a). Hence, [^125^I]**26a** represents an early example of an ABP. Further investigations by Mason et al. led to the development of radiotracer [^125^I]**26b**, in which the Z group of [^125^I]**26a** has been replaced by Fmoc and a second iodo-substituent has been attached *ortho* to the hydroxyl group in the tyrosine side chain (Xing et al., 1998). Notably, the non-radioactive reference compound **26b** showed an inhibitory potential toward cathepsin B that was 15-fold higher than that of its deiodinated counterpart and more than twice as strong compared to the corresponding monoiodinated derivative. Considering inhibition of cathepsin L, the influence of iodination was inverse compared to cathepsin B, while inhibition of cathepsin L by **26b** was still faster than that of cathepsin B. **26b** was devoid of inhibitory potency toward cathepsin S. Radioiodination of deiodo-**26b** in the presence of non-radioactive sodium iodide resulted in the formation of [^125^I]**26b**. Thus, [^125^I]**26b** represents a carrier-added radiotracer in contrast to non-carrier-added [^125^I]**26a**. This is reflected by the specific activity of [^125^I]**25b**, which is with 8.3 MBq/μmol considerably lower than that of [^125^I]**26a**. Incubation of [^125^I]**26b** with a panel of human breast tumor cells and subsequent cell lysis and electrophoretic protein separation by SDS-PAGE showed that these cells contain up to four different molecular species of cathepsin L and two different forms of cathepsin B. The knowledge of the radiotracer's specific activity enabled the calculation of quantitative amounts of the different cathepsin B and L species in these cells in pmol/mg of cellular protein. Interestingly, these studies have revealed that higher levels of active cathepsins B and L are expressed in invasive breast tumor cells (MDA-MB-231, MDA-MB-435S and HS-578T), compared with those that are not invasive (MCF7 and SK-BR-3).

A diazoketone warhead is also contained in the activity-based probe [^125^I]**27** (Falgueyret et al., 2004). Its labeling with iodine-125 has been done in a manner that is independent of the presence of hydroxyphenyl groups, which will be explained more in detail below. Non-radioactive **27** has been characterized as a potent inactivator of the cathepsins B, K, L and S. Its potency is ranked in the order K>L≈S>B. This inhibition profile allowed to use radiolabeled [^125^I]**27** for the identification of the pairs of cathepsins B/L and B/K in human HepG2 hepatoma cells and rabbit HIG82 synoviocytes, respectively, and cathepsin S in the Ramos B lymphoma (human Burkitt's lymphoma) cell line, which has been done by incubating the probe with the corresponding cell lysates and subsequent electrophoretic separation by one and two-dimensional SDS-PAGE. The ^125^I-labeled enzyme-inhibitor complexes were detected autoradiographically and the results have been verified by Western blotting using specific antibodies for the individual cathepsins. [^125^I]**27** was also used to determine the *in cellulo* efficiency of cathepsin inhibitors in so called whole-cell enzyme occupancy assays. In such assays, cells are preincubated with the inhibitor of interest at varying concentrations for a defined time followed by incubation with the radiolabeled probe. Subsequent cell lysis and separation as outlined above followed by measuring the radiographic density of the electrophoretic spots and data analysis provide the cellular IC_50_-values for distinct cathepsins. As the authors noted, to obtain meaningful values for reversible inhibitors, the assay has to be performed under conditions in which the irreversibly binding probe does not displace the inhibitor from the enzyme. Therefore, careful optimization of the incubation time with the radiolabeled probe is required. By applying the methodology outlined above, radiotracer [^125^I]**27** has been also used to identify cathepsin S in blood *in vitro* and to determine the occupancy of cathepsin S by a dipeptide-derived nitrile closely related to odanacatib in blood of rhesus monkeys *ex vivo* (Veilleux et al., 2011).

The development of ^125^I-labeled radiotracers for cysteine cathepsins based on irreversible inhibitors different from diazoketones was pursued by Bogyo et al. (2000). The lead structures for tracer design were the epoxysuccinyl derivatives JPM-565 (**7a**), CA074 (**9a**) and the dipeptide-derived phenyl vinyl sulfone LHVS (**5**). These compounds have been selected because **7a** offers the opportunity of promiscuous targeting of several cysteine cathepsins such as L, B, and S, whereas **9a** and **5** are selective inactivators of cathepsin B and cathepsin S, respectively. To allow the facile introduction of radioiodine, the leads had to be equipped with hydroxyphenyl moieties. For the vinyl sulfone **5**, this was achieved by attachment of a hydroxyl group to the phenyl residue *para* to the sulfonyl group resulting in deiodo-**28**. In the case of CA074 (**9a**) the N-bound propyl chain was replaced by a 2-(4-hydroxyphenyl)ethyl residue. This structural change slightly attenuated the inhibitory activity but the selectivity for cathepsin B was retained. The non-radioactive tracer compound **30a** has not been evaluated kinetically but iodination has been judged to be tolerable to the enzyme-inhibitor interaction on the basis of molecular docking. The *in cellulo* evaluation of the radiotracers [^125^I]**28**, [^125^I]**29a** and [^125^I]**30a** has been performed in the dendritic cell line DC2.4, the human pro-monocyte cell line U937 and cytotrophoblasts derived from human placenta in a similar fashion as in the works described above. Only [^125^I]**30a** was able to visualize specifically cathepsin B on the radioelectropherograms. The specificity of this radiotracer toward cathepsin B was further confirmed by comparing lysates of splenocytes from wildtype mice or mice in which the cathepsin B, S, or L gene had been deleted using radio-SDS-PAGE. The radiotracers [^125^I]**29a** and [^125^I]**30a** were used to analyze the levels of active cysteine cathepsins in homogenates of patient-derived neoplastic tissues obtained from normal, primary and metastatic tumors. The corresponding radioelectropherograms indicated an increase in cathepsin B labeling in metastatic tissue relative to primary tumors and normal tissue, even though the extent of upregulation differed among the individuals. In contrast, the intensity of the cathepsin S band was lower in the tumor samples. Invasion of cells to other tissues is part of normal developmental processes of organisms. An example is the migration of cytotrophoblasts to the uterus during the development of the human placenta. Differently to tumor metastasis, these processes are strictly regulated. The differentiation of cytotrophoblasts toward the invasive phenotype was stimulated by the presence of ECM-mimicking matrigel and the active cysteine cathepsins in the lysates were detected by [^125^I]**29a** and [^125^I]**30a** and radio-SDS-PAGE. The presence of cathepsin S and B increased over the time of matrigel exposure. This nicely illustrates how radiotracers that covalently interact with enzymes can be used to shed light on their role in biological processes with the help of proteomics-related approaches.

The probes [^125^I]**31** and [^125^I]**32** combine the ^125^I-radiolabel with a biotinyl tag as affinity label to enable detection of the enzyme-inhibitor complexes by two independent modalities (Greenbaum et al., 2000). Both radiotracers are derived from E64 (**6**), whose agmatine moiety has been replaced by a tyrosyl lysine amide to obtain deiodo-**31**. Inserting an aminohexanoic spacer between the tyrosine and lysine residues resulted in deiodo-**32**. Similar as for [^125^I]**30**, which retained the selectivity of CA074 (**9a**) toward cathepsin B, [^125^I]**31** and [^125^I]**32** exhibited pan-reactivity against lysosomal cysteine cathepsins similar to their parent compound E64 (**6**). The probes have been evaluated similar as the other ^125^I-labeled epoxysuccinyl derivatives discussed above. Their labeling profiles in radio-SDS-PAGE were similar to that of [^125^I]**29a**. In addition, the labeling profiles of deiodo-**31** and deiodo-**32** were determined by Western blotting using an avidin-horseradish peroxidase conjugate. In the range of 20–40 kDa the band patterns for both detection modes are consistent, whereas streptavidin-based detection exhibited additional bands in the range above 40 kDa. This finding has been mainly attributed to the presence of endogenously biotinylated proteins in the cell lysates. [^125^I]**32** has been used to perform facile selectivity profiling of inhibitors in the presence of cell lysates. A library of derivatives of deiodo-**32** has been synthesized where the leucine residue has been replaced by all other proteinogenic amino acids except for cysteine and methionine, which was replaced by norleucine. Their selectivity profiles have been evaluated by addition of each inhibitor to DC2.4 lysates at a concentration of 50 μM prior to addition of [^125^I]**32** and subsequent simultaneous electrophoretic separation. Furthermore, deiodo-**32** has been used for the identification of cysteine cathepsins in rat kidney tissue homogenates. Fractionation by anion exchange chromatography guided by SDS-PAGE was followed by concentration of the pooled fractions of interest using an avidin column. The concentrated fractions were subjected to two-dimensional SDS-PAGE and the excised and trypsin-digested spots were analyzed by mass spectrometry. The results unambiguously identified the cathepsins B, H and L and thus confirmed these enzymes to be the major cysteine proteases in kidney tissue. The application of [^125^I]**32** and deiodo-**32** for radioactivity measurements and mass spectrometry as independent detection modes demonstrate the versatility of these dual-label probes. [^125^I]**32** has further been used in defining biological functions of papain-like cysteine proteases, such as the role of cathepsin L as alternative prohormone convertase to liberate the peptidic neurotransmitter enkephalin in the brain (Yasothornsrikul et al., 2003) and elucidation of the function of parasitic cysteine proteases in invasion of host tissues (Greenbaum et al., 2002; Dvorak et al., 2009).

To create such dual-label probes which enable to address a broad range of cysteine proteases such as various clan CD enzymes (caspases, legumain and the bacterial gingipains), the acyloxymethylketone-based probes [^125^I]**33a**-**f** were developed (Kato et al., 2005). Upon varying the residue in P^1^, the different proteases could be visualized by radio-SDS-PAGE. When leucine was introduced as P^1^ amino acid, the CA enzyme cathepsin L was also efficiently labeled in the radioelectropherograms.

Peptide-derived acyloxymethylketones were also the basis for the design of the cathepsin B-targeting radiotracers [^125^I]**34a** and **b** (Figure 10) (Edem et al., 2014). The dipeptide portion consists of the Phe-Lys scaffold and the ^125^I-carrying groups are attached via the lysine side chain. Regarding the introduction of radioiodine a more sophisticated approach has been chosen than for the radiotracers discussed so far. The electrophilic iodination is done on an aromatic group containing an electrofuge differing from a proton which implicates the advantage of independence from a neighboring +M substituent. Such electrofuges are typically metallyl groups of group 13 and 14 elements, with trialkylstannyls being the most commonly used ones (Kabalka and Varma, 1989). Generally, groups of this type, which facilitate the introduction of the radionuclide, are called prosthetic groups. In the case of radioiodination the use of prosthetic groups can have direct implications on pharmacokinetics. Iodine radioisotopes attached at the 3-position of 4-hydroxyphenyl moieties can be removed by the action of deiodinases, enzymes that participate in the metabolism of thyroid hormones. Because enzymatic aromatic deiodination is impeded in the absence of the hydroxy group, moieties that result in labeled *meta*- and *para*-iodobenzoyl groups are suitable for the metabolically stable introduction of radioiodine (Mier and Nissen, 2010). However, the introduction of such prosthetic groups into protein-binding ligands can potentially impede their binding affinity due to their increased steric demand. This was observed in the study of Edem et al. when the 4-iodobenzoyl group was directly attached to the ε-amino group of the lysine side chain as the second-order rate constants *k*_inact_/*K*_I_ for inactivation dropped by 50-fold compared to the unmodified parent compound. When the iodo-substituent was introduced in the *meta*-position, the loss in inhibitory activity was even stronger. The insertion of an aminohexanoic linker between *N*^ε^ and the iodobenzoyl group did not result in restored inhibitory potency, but when the spacer length was extended from a pentamethylene to a three-unit poly(ethylene glycol) (PEG) chain the inactivation rates approached the range of the parent compound, again with *meta*-iodo substitution resulting in less efficient inhibition than *para*. Both **34a** and **b** were synthesized in their ^125^I-labeled versions. For this purpose, their corresponding tris(tridecafluorooctyl)arylstannanes were prepared and reacted with sodium [^125^I]iodide in the presence of iodogen at room temperature under slightly acidic conditions. The tin-bound fluoroalkyl groups allow separating the radiotracer products from the excess of precursor by simple fluorous solid-phase extraction, to provide [^125^I]**34a** and **b** in high radiochemical purities and specific activities of >23 GBq/μmol. The ability of both radiotracers to target cathepsin B has been demonstrated by radio-SDS-PAGE analysis of the ^125^I-labeled enzyme-inhibitor complex. Due to the prolonged incubation time of 1 h, also the less potent inhibitor [^125^I]**34a** was capable of cathepsin B labeling to a similar extent as [^125^I]**34b**. The labeled bands did not appear when the radiotracers were incubated with cathepsin B in the presence of CA074 (**9a**). To obtain information regarding their tumor-targeting ability, [^125^I]**34a** and **b** were investigated in *ex vivo* biodistribution studies in immunodeficient mice bearing tumor xenografts derived from the human MDA-MB-231 breast cancer cell line. The distribution of the ^125^I activity across the different organs including the tumor was determined at time points 0.5, 5, and 23 h p.i. An increased uptake of activity in the thyroid after 5 h p.i. indicates the metabolic release of [^125^I]iodide from both radiotracers. The tumor/muscle ratio for [^125^I]**34b** steadily increased over time and reached a value of 7.3 after 23 h p.i., which is significantly higher than that for [^125^I]**34a**. This result is in agreement with the higher inhibitory potency of [^125^I]**34b**. The liver uptake of [^125^I]**34b** was approximately 5-fold reduced compared to that of [^125^I]**34a**, which has been attributed to the hydrophilicity that is conferred by the three-unit PEG linker. Therefore, the study by Edem et al. (2014) provides for the first time data on the *in vivo* behavior of a ^125^I-labeled cathepsin inhibitor, whereas the characterizations of the other radiotracers discussed so far were focused on their *in vitro* application.

#### Radiotracers based on fluorine-18, radiocarbon isotopes and tritium

Besides iodine-125, radiopharmaceutically relevant radioisotopes can be found for each element within the halogen group of the periodic table (Adam and Wilbur, 2005; Wuest, 2005; Engle et al., 2011). In regard to PET, fluorine-18 is certainly the most important one as it has favorable properties such as high content of positron emission (97%) and intermediate half-life of 109.8 min (Table 1). Its rather long half-life—compared to other non-metallic PET nuclides—enables multi-step syntheses within one to three half-lives. Because furthermore fluorine forms very stable covalent bonds with carbon, this radiohalogen is unparalleled in being used for the development of small molecule-based PET tracers (Miller et al., 2008; Cole et al., 2014).

**Figure 11:**
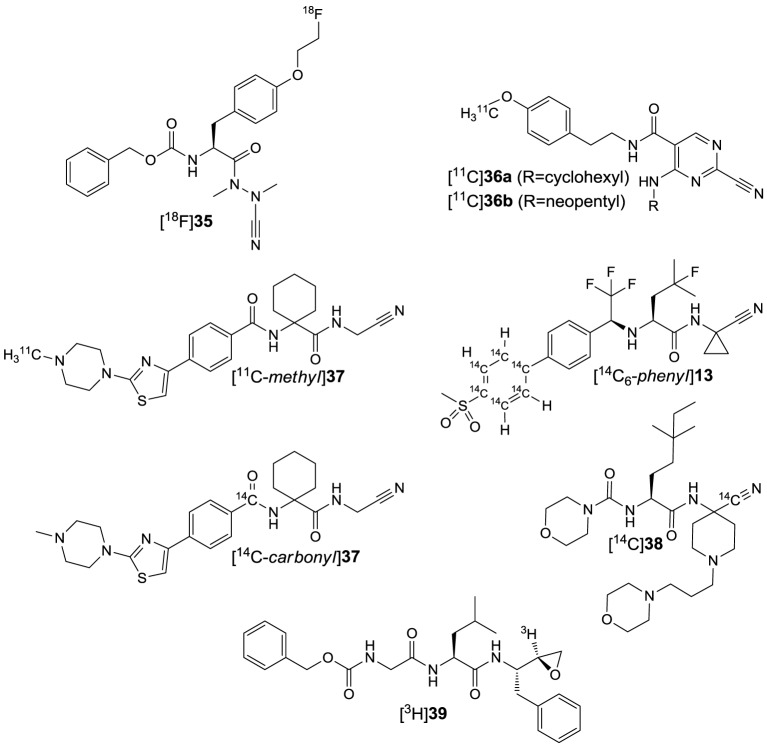
**Cysteine-cathepsin targeting radiotracers labeled with “organic radionuclides**.”

Compound [^18^F]**35** (Figure 11) represents the first ^18^F-labeled radiotracer targeted against cysteine cathepsins (Löser et al., 2013). This molecular probe is based on an inhibitor of the azadipeptide nitrile chemotype. Azadipeptide nitriles have been characterized as highly potent toward papain-like cysteine proteases and stable against the degradation by other proteases (Löser et al., 2008; Frizler et al., 2011; Yang et al., 2012). To enable the convenient introduction of fluorine-18, the tyrosine residue in P^2^ position was etherified with a fluoroethyl chain. The non-radioactive reference compound **35** exhibited high binding affinities toward the oncologically relevant cathepsins B, L, S, and K. The *K*_i_-value for cathepsin B was 2.4 nM, whereas those toward cathepsin L, S, and K were in the subnanomolar range. The process of ^18^F-labeling to obtain the corresponding radiotracer [^18^F]**35** was established as a two-step, one-pot radiosynthesis. In this procedure [^18^F]fluoride was converted with ethylene glycol-1,2-dinosylate to 2-[^18^F]fluorethyl nosylate which was reacted without isolation with the corresponding phenolic precursor. This approach resulted in higher radiochemical yields for [^18^F]**35** than the use of 2-[^18^F]fluoroethyl tosylate, which is the commonly employed agent for ^18^F-fluoroethylations (Zhang and Suzuki, 2007). The developed procedure was also superior to the direct conversion of corresponding benzenesulfonate precursors with [^18^F]fluoride. The well-established ^18^F-labeling process allowed the extensive radiopharmacological characterization of [^18^F]**35** in immunodeficient mice bearing xenografted tumors derived from the human lung carcinoma cell line NCI-H292. Immunohistochemical investigations confirmed the presence of the targeted cathepsins in the neoplastic tissue. Radiotracer [^18^F]**35** was studied for its *ex vivo* biodistribution and pharmacokinetics and stability *in vivo* in rats and mice. Slow blood clearance of the ^18^F-activity results from conjugate formation between [^18^F]**35** and glutathione and retention of the formed metabolite in the erythrocytes, which has been confirmed by *in vitro* experiments. Despite rapid biotransformation, approximately 35% of the original radiotracer was still detectable in the blood activity fraction at 60 min p.i., which suggests that it should be available to the tumor-associated cysteine cathepsins. Accordingly, the tumor/muscle and tumor/blood ratios in the NCI-H292 carrying mice as determined by dynamic PET imaging were slowly increasing with tumor/muscle ratios reaching values significantly greater than three at 120 min p.i. This can be considered as indicative of a specific tumor accumulation (Löser et al., 2013). Therefore, despite unfavorable pharmacokinetic behavior due to inherent thiol reactivity, azadipeptide nitriles seem to be capable of tumor targeting.

Cathepsin-targeting radiotracers based on cyano group-containing inhibitors have been also developed by Rodnick et al. (2014). In difference, the motivation of this study was the visualization of cathepsin K activity in osseous tissue, the inhibitor scaffold was non-peptidic and the chosen radiolabel was the positron emitter carbon-11 instead of fluorine-18 (Table 1). The choice of the radiolabel was influenced by the fact that ^18^F-labeled tracers can potentially undergo *in vivo* defluorination and the released inorganic fluoride accumulates in the skeleton, which could have made judgements about the specific targeting of cathepsin K in bone tissue difficult. The selected inhibitors represent the 2-cyanopyrimidine chemotype of cysteine cathepsin inhibitors (Teno and Masuya, 2010) with **36a** and **b** (Figure 11) exhibiting IC_50_-values toward cathepsin K of 22 and 3 pM, respectively, and a 10–1000 fold selectivity over the cathepsins L and S (Altmann et al., 2007). Labeling with carbon-11 for both cyanopyrimidines was performed by ^11^C-methylation of the corresponding phenolic precursors with [^11^C]methyl iodide generated by gas-phase reduction of cyclotron-produced [^11^C]carbon dioxide (Pretze et al., 2011). The *in vivo* radiopharmacological evaluation of [^11^C]**36a** and **b** has been performed by dynamic PET imaging in rats. The obtained images indicated an accumulation of ^11^C activity in the distal ulna, distal femur and proximal tibia, among other regions where bone remodeling under the involvement of cathepsin K is occurring. The uptake of [^11^C]**36b** in these regions was 2–3 fold higher than in muscle tissue and could be attenuated by preinjection of non-radioactive **36b**. In accordance with this result, the uptake for [^11^C]**36a** of high specific activity (414 GBq/μmol) was higher than that for [^11^C]**36a** of low specific activity (10.8 GBq/μmol, corresponding to co-injection of 14 μg/kg of non-radioactive **36a**) (Rodnick et al., 2014). These results support the suitability of [^11^C]**36a** and **b** as radiotracers to image the activity of cathepsin K *in vivo*.

Imaging of cathepsin K is also in the focus of the study by Bennacef et al. (2013) for which only preliminary results have been published yet in abstract form. The dipeptide-derived nitrile **37** containing homocycloleucine in P^2^ is a potent inhibitor of cathepsin K with an IC_50_ of 0.2 nM (Falgueyret et al., 2005). [^11^C]**37** has been synthesized by ^11^C-methylation of *nor*-**37** using [^11^C]methyl triflate in a specific activity of 1229 GBq/μmol. PET imaging studies of [^11^C]**37** in juvenile rhesus monkeys indicated an increased uptake in actively growing bone regions (SUV's ≈2.0 for distal femur and proximal tibia vs. ≈0.7 for mid-femur; SUV: standardized uptake value). Uptake of [^11^C]**37** was attenuated upon pretreatment with a different inhibitor, which is selective for cathepsin K (Bennacef et al., 2013).

Historically, carbon-11 has been used in biochemical research many years before PET has been invented and before long-life carbon-14 was available in larger scale, such as [^11^C]CO_2_ for early studies on CO_2_ assimilation in barley plants (Ruben et al., 1939) or to investigate its incorporation into glycogen in starved rats (Solomon et al., 1941). Later, carbon-14 became the preferred carbon radioisotope for *in vitro* investigations (Kamen, 1963, 1986), which culminated in the elucidation of the metabolic cycle of the photosynthetic dark reaction (Calvin, 1962). Until today, ^14^C-labeled compounds are of great importance to gain insight into the ADME properties of drug molecules (Elmore, 2009; Isin et al., 2012). Such studies are integral part of clinical drug development and the results are only occasionally published. Considering the variety of cathepsin inhibitors that are in clinical development, the collection of ^14^C-labeled tracers related to cysteine cathepsins in Figure 11 is certainly not complete. Contrary to radiotracers based on short-lived nuclides, the specific activity of ^14^C-tracers is restricted to much lower values. However, because long-lived radionuclides allow the incorporation of multiple radionuclides per tracer molecule, this limitation can be partly counterbalanced. This concept has been realized in [^14^C_6_-*phenyl*]**13**, a ^14^C-labeled odanacatib (Kassahun et al., 2011). The radiotracer has been obtained in a specific activity of 2.9 MBq/μmol and was investigated in rats, dogs, and rhesus monkeys for its metabolite profile. The biotransformation pathways are different in these species. In rats and dogs the main route is hydroxylation of the sulfone methyl group followed by spontaneous de-hydroxymethylation to the corresponding sulfinic acid, which is either excreted or undergoes oxidation to the sulfonic acid. In difference, the main metabolic route in monkeys is initiated by oxidation at one of the methyl groups in the P^2^ side chain, which is followed by further oxidation or *O*-glucuronidation. The biotransformation of [^14^C_6_-*phenyl*]**13** observed in monkeys was similar in humans, while the content of excreted unmodified compound is higher in men. In accordance with the preclinical results from animals, biliary excretion is predominant over renal elimination and no metabolites are retained in the circulation (Kassahun et al., 2014).

Besides ADME studies in humans, ^14^C-labeled compounds are typically used in autoradiography and whole-body biodistribution studies in small animals. In this context, [^14^C]**37** has been prepared in a two-step synthesis consisting of conversion of 4-(2-(4-methylpiperazin-1-yl)thiazol-4-yl)phenyl)lithium with [^14^C]CO_2_ followed by amide bond formation with the corresponding deprotected dipeptide nitrile. The *ex vivo* biodistribution studies in rats have shown that the levels of [^14^C]**37** or its metabolites in the liver, spleen, lung and kidney are 6–12 times higher than in the blood. These results are interpreted to reflect the lysosomotropic character of **37**, which is conferred by the moderately basic piperazine moiety, because the high-uptake organs are composed of cells that are rich in lysosomes. This interpretation is in agreement with the high volume of distribution, which has been determined to be 11 L/kg for [^14^C]**37** (Falgueyret et al., 2005). Lysosomotropism has been identified as the main reason for adverse side effects observed during the clinical development of the cathepsin K inhibitor balicatib (Brömme and Lecaille, 2009). The dipeptide nitrile **38** is a potent and selective inhibitor of cathepsin S developed by Boehringer Ingelheim (Lorenz et al., 2010). To obtain information on its ADME properties, introduction of carbon-14 was achieved via Strecker reaction with sodium [^14^C]cyanide. The final product [^14^C]**38** was obtained in a specific activity of 1.8 MBq/μmol. Apart from details on the radiosynthesis, no radiopharmacological results are disclosed (Latli et al., 2012).

Another radionuclide with similar properties is tritium. In contrast to carbon-14 it has a much shorter half-life of 12.32 years and is therefore less problematic regarding radiation protection issues. However, the mean energy of the electron emitted upon disintegration of a tritium nucleus is only about 10% of that for the ^14^C-borne electrons (Table 1), which means that the β^−^ particles emitted from tritium are almost completely shielded by the wall of the reaction vessel. This implicates the use of liquid scintillation counting or radioluminographic methods for the detection of ^3^H activity. Other potential drawbacks of labeling with tritium are the biological instability of the resulting radiotracer and kinetic isotope effects, when the chemical process to be studied involves the labeled position (Penner et al., 2012). However, labeling of organic molecules with tritium is often facile to achieve by methods such as exposure of compounds to high activities of tritium gas (known as the Wilzbach method), catalyzed ^1^H/^3^H isotope exchange or introduction by reaction with tritiated building blocks, for example reduction of carbonyl compounds with the tritium-based versions of complex hydrides (Saljoughian, 2002; Voges et al., 2011). The latter strategy was pursued by Albeck and Kliper for labeling of the *N*-tripeptidyl-α-aminoalkyl epoxide **39** with ^3^H. Reduction of the corresponding tripeptidyl bromomethyl ketone with sodium borotritide and subsequent cyclization afforded epoxide [^3^H]**39** in a specific activity of 833 MBq/mmol. Papain was incubated with equimolar amounts of [^3^H]**39**. Separation of the labeled enzyme-inhibitor complex by dialysis and determination of the ^3^H activity by liquid scintillation counting indicated that 94% of the enzyme associated with [^3^H]**39**. When this experiment was performed with papain that was pretreated with the thiol-reactive reagent DTNB or heat-denatured papain, only 9% and less than 0.5% of [^3^H]**39**, respectively, associated with the enzyme. On the basis of these results the authors concluded that *N*-peptidyl-α-aminoalkyl epoxides interact in a 1:1 stoichiometry with the active-site cysteine residue in papain-like cysteine proteases and that this interaction is dependent on the catalytic competence of the enzyme (Albeck and Kliper, 1997). This example may serve to illustrate the expedience of radiolabeled irreversible enzyme inhibitors to determine the stoichiometry of enzyme inactivation.

#### Radiometal-based tracers

**Figure 12:**
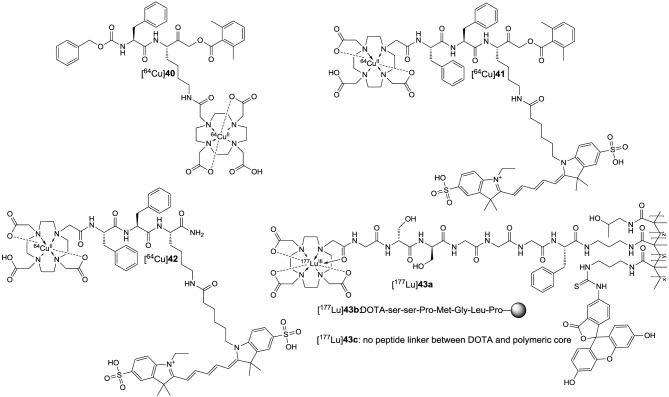
**Radiometal-based cysteine-cathepsin targeting radiotracers**.

Most of the known radionuclides are of metallic character, which is simply due the fact that the majority of the chemical elements are metals. Many among them are valuable for SPECT and PET imaging (Wadas et al., 2010). A well-studied metallic PET nuclide is copper-64 (Table 1). Compared to fluorine-18, it offers the advantage of extended half-life and coordinative bond formation under rather mild conditions. However, the latter advantage implicates the drawback of modification with spatially demanding chelators for radiotracer design, which potentially can compromise target interaction, especially in the case of small molecules. The ^64^Cu-labeled acyloxymethyl ketones shown in Figure 12 have been the first reported PET tracers which target cysteine cathepsins (Ren et al., 2011). The design of compound [^64^Cu]**40** was guided by the structure of GB123, a fluorescent AOMK capable of cathepsin B and L optical imaging^7^. Its Cy5 fluorophore has been replaced by the macrocyclic chelator DOTA which has been connected via an amide bond with one of its carboxymethyl groups to the *N*^ε^ of the lysine in P^1^ to result in the labeling precursor of [^64^Cu]**40**. To enable equipment with a copper-64 and fluorescent label, the Z-group was replaced by phenylalanine and the chelator relocated from the lysine side chain to the N-terminus, which provided the vacancy to attach a Cy5 label (precursor for [^64^Cu]**41**). To be able to compare the activity-based probe [^64^Cu]**41** with a substrate analog as control, its corresponding primary amide [^64^Cu]**42** was prepared. Labeling with copper-64 was done by incubating the chelator-functionalized precursors with [^64^Cu]CuCl_2_ in aqueous solution at pH 5.5 and 50°C to obtain the corresponding radiotracers in high radiochemical purity and specific activities ranging between 1.1 and 17.8 GBq/μmol. The whole-body biodistribution of the three ^64^Cu-labeled probes was studied in athymic nude mice bearing subcutaneously grafted tumors derived from the human breast cancer cell line MDA-MB-435 and the oncogenically transformed murine C2C12/Ras myeloblastoma line. The tumor uptake of [^64^Cu]**40** at 24 h p.i. was significantly higher in the C2C12/Ras tumors, which correlates with a higher cysteine cathepsin activity in this cell line compared to MDA-MB-435. The difference in the absolute uptake is also reflected in differing tumor/muscle ratios. While the radiotracer exhibited a low uptake in most non-tumor tissues, activity accumulation in the liver was high. This observation has been interpreted to result from de- or transchelation of the ^64^Cu-copper complex, which is in accordance with results that render DOTA suboptimal for stable complexation of Cu^2+^ ions (Maheshwari et al., 2012; Cai and Anderson, 2014; Price and Orvig, 2014; Zarschler et al., 2014). Interestingly, the uptake of the bimodal probe [^64^Cu]**41** in the C2C12/Ras tumors was more than 10 times higher than that of [^64^Cu]**40** with a tumor/muscle ratio of 13 at 24 h p.i. This phenomenon might be due to the amphiphilic character conferred by the Cy5 moiety. Also the uptake of the corresponding substrate [^64^Cu]**42** was higher compared to [^64^Cu]**40**, but considerably lower than that of its AOMK analog [^64^Cu]**41**. Notably, the tumor/muscle ratio at 2 h p.i. for the substrate [^64^Cu]**42** was in the same range as that for the irreversible inhibitor [^64^Cu]**40**. μPET investigations mainly confirmed the results of the biodistribution study. PET imaging included also the investigation of the bimodal AOMK-based probe [^64^Cu]**41** in mice bearing murine 4T1 tumors with high cysteine cathepsin expression. Subsequent to the end point of imaging at 24 p.i., the cathepsin activity was investigated in tumor homogenates for all three cell lines by SDS-PAGE. This was performed both by quantifying the fluorescence originating from the [^64^Cu]**41**-cathepsin complexes as well as by analyzing the residual cysteine cathepsin activity upon addition of the fluorescence-only probe GB123 to the tumor homogenates. For both methods the measured in-gel fluorescence signals correlated well with the tumor/muscle ratios determined by PET imaging. Furthermore, the tumor uptake of [^64^Cu]**41** was reduced by more than factor 2 upon pretreatment with large amounts of **4**, a vinyl sulfone-based broad spectrum inhibitor of cysteine cathepsins, in mice carrying tumors derived from the human breast cancer cell line MDA-MB-231MFP. In conclusion, the work of Ren et al. established very well that the intratumoral imaging signals of [^64^Cu]**41** are associated with the cysteine cathepsin activity. Moreover, the superiority of [^64^Cu]**41** over [^64^Cu]**40** demonstrates that the conjugation of radiotracers with fluorophores may improve their pharmacokinetic properties in addition to providing the opportunity of bimodal imaging.

Besides *in vivo* diagnostics, molecules containing radionuclides are of potential interest for targeted radiation therapy of tumors. In particular, this applies to nuclides which emit charged energetic particles such as β^−^ and α particles or Auger and conversion electrons. Preferably, their half-life should be in the range of a few days until several weeks, and the nuclear transformation should be accompanied by the emission of γ photons with an energy range between 70 and 360 keV, to enable tracking of the agents by SPECT imaging (Pouget et al., 2011; Cutler et al., 2013). A nuclide that matches these criterions quite well is the radiolanthanide lutetium-177 (Table 1). With a maximum energy of 498 keV corresponding to a mean range of 670 μm for the emitted β^−^ particle it is almost ideally suited to treat locally confined primary and metastatic lesions with limited radiation exposure of the surrounding normal tissue (Zalutsky, 2011). Regarding its chemical properties, lutetium prefers the oxidation state +3 and high coordination numbers with 9 being the most encountered one. This makes DOTA a suitable chelator for Lu^3+^ ions. In the resulting complexes, eight coordination sites are occupied by the ligator atoms of DOTA and an additional is probably provided by a water molecule. ^177^Lu-DOTA complexes conjugated to bone-seeking bisphosphonates, analogs of somatostatin and bombesin as well as a monoclonal antibody targeting the prostate-specific membrane antigen have been evaluated in clinical trials for the treatment of metastatic prostate cancer and neuroendocrine tumors (Cutler et al., 2013). Apart from this targeted approach, conjugation of such radiometal complexes to polymers of sufficient molecular mass and favorable properties can be an alternative to deliver the radiation dose to the tumor by taking advantage of the enhanced permeability and retention (EPR) effect. This effect is mainly caused by a fenestrated endothelium in the tumor blood vessels on the one side and defective lymphatic drainage on the other. Therefore, macromolecules with a molecular mass above a threshold around 20 kDa accumulate in the tumor tissue, which is also referred to as passive targeting. A neutral charge and a molecular mass greater than 30–50 kDa will further facilitate tumor delivery of the polymer due to reduced renal filtration (Haag and Kratz, 2006). However, with increasing mass the chance of phagocytotic uptake by cells of the reticuloendothelial system, especially macrophages, rises. Because these cells reside mainly in the spleen and the liver, toxic effects to these non-target organs have to be taken into account for therapy with radionuclide-carrying polymers. To tackle this problem, Ogbomo et al. (2013) investigated the effect of conjugation of ^177^Lu-DOTA complexes to *N*-(2-hydroxypropyl)methacrylamide-based copolymers via cathepsin-cleavable linkers on the clearance from liver and spleen. The structure of the polymeric constructs is schematically depicted in Figure 12. Their synthesis has been achieved by copolymerizing the monomeric building blocks *N*-(2-hydroxypropyl)methacrylamide and the DOTA-peptide/FITC-functionalized *N*-(3-aminopropyl)methacrylamides in a molar ratio of 98:1:1, respectively. Therefore, the resulting copolymers represent bimodal probes because one half of the (3-aminopropyl)methacrylamide was conjugated to the chelator and the other to fluoresceine. The peptide sequences (Gly)_3_-Phe (contained in [^177^Lu]**43a**) and Pro-Met-Gly-Ile-Pro (contained in [^177^Lu]**43b**) were chosen as linkers to mediate cleavage by the cathepsins B and S, respectively. This selection was based on literature reports. In both cases the tripeptide Gly-(d-Ser)_2_ was placed between the N-terminus and the DOTA macrocycle as linker to facilitate clearance. A construct lacking the peptidic linker between the DOTA moiety and the polymeric core serves as control vehicle ([^177^Lu]**43c**). To prove the susceptibility of the peptides to cathepsin-catalyzed hydrolysis, the isolated demetallated DOTA-conjugated peptides derived from **43a** and **b** were investigated for their degradation upon incubation with isolated cathepsin B and S, respectively. Cleavage of the peptide contained in **43a** by cathepsin B (~0.9 μM) proceeded with a half-life of approximately 24 h while degradation of the peptide included in **43b** catalyzed by cathepsin S (~1.0 μM) was much slower. The cleavage occurred after the second glycine residue in **43a** and between glycine and leucine in **43b**, as confirmed by mass spectrometry. Accordingly, the ^177^Lu-labeled polymeric vehicles [^177^Lu]**43a** and **b** are cleaved by the respective cysteine cathepsins, while all three polymers [^177^Lu]**43a**-**c** were stable in serum for 72 h. To assure that tumor targeting relies exclusively on EPR, the uptake of the demetallated copolymers was studied by FACS analysis in the human pancreatic adenocarcinoma cell line HPAC and macrophages. While a time-dependent increase in cell-associated fluorescence was measureable in macrophages for all three compounds, virtually no increase was observed for the tumor cells within the same range. The activities of cathepsin B and S in the HPAC cells were 70 and 10-fold lower than in macrophages, respectively. *In vivo* evaluation of ^177^Lu-containing polymers has been performed in SCID mice bearing xenografted tumors derived from the HPAC cell line. The *ex vivo* whole-body biodistribution of [^177^Lu]**43a**-**c** was investigated at 24 and 72 h p.i. At 24 h p.i. the liver uptake of [^177^Lu]**43a** and **b** was lower than that for [^177^Lu]**43c**, while the values for [^177^Lu]**43b** were in between those for [^177^Lu]**43a** and [^177^Lu]**43c**. From 24 until 72 h p.i. the liver uptake of the non-cleavable control [^177^Lu]**43c** increased 1.5-fold, whereas the level of cathepsin-responsive [^177^Lu]**43a** and **b** remained constant. The situation proved to be similar in the spleen except that the level of [^177^Lu]**43c** increased even more from 24 to 72 h p.i. The tumor/spleen ratios reached after 72 h were more favorable for the cleavable analogs [^177^Lu]**43a** and **b**. Unfortunately, their absolute tumor uptakes compared to stable [^177^Lu]**42c** were lower by factors of 3 and 2, respectively. Despite the reasons for this result remained unclear, enhanced blood retention and potential tumor metabolism of [^177^Lu]**43a** and **b** might be possible explanations. In this context it should be mentioned, that similar approaches exploited protease-cleavable linkers for selective tumor delivery of therapeutic agents by taking advantage of the tumor-associated protease activity (Gill and Loadman, 2008).

## Concluding remarks

In the more than 80 years that have been passed since the discovery by Willstätter and Bamann, the field of cysteine cathepsins has seen a tremendous development in regard to the elucidation of their structures and physiological functions in the last two decades. Especially their roles in tumor progression and metastasis, but also in etiology, manifestation and progression of other diseases are more and more recognized in detail. Molecular probes contributed to this process to a substantial extent and will help to define the pathophysiological functions of these enzymes even more precisely in the future. The use of radiotracers for functional *in vivo* imaging of cathepsins has just been initiated in the last years and will substantially facilitate the clinical translation of inhibitors.

In this article we intended to provide an overview on recently developed fluorescent and radioactive molecular probes targeted against cysteine cathepsins. In addition, we hope that we rendered the field of probe development palatable to the medicinal and pharmaceutical chemist, as it combines many exciting and diverse facets of chemistry to study intriguing biological processes, not only in the context of cathepsins and tumor biology.

### Conflict of interest statement

The authors declare that the research was conducted in the absence of any commercial or financial relationships that could be construed as a potential conflict of interest.

## Footnotes

^1^http://merops.sanger.ac.uk/cgi-bin/clansum?clan=CA.

^2^http://merops.sanger.ac.uk/cgi-bin/smi_summary?mid=J10.011.

^3^A Study to Examine the Effects of an Experimental Drug on Women With Breast Cancer and Metastatic Bone Disease (MBD)(0822-016)(COMPLETED), https://clinicaltrials.gov/ct2/show/NCT00399802.

^4^A Study to Assess the Effects of MK0822 in Reducing the Risk of Bone Metastasis in Women With Breast Cancer (0822-029), https://clinicaltrials.gov/ct2/show/NCT00692458.

^5^A Phase III Study to Assess the Safety, Tolerability, and Efficacy of MK0822 (Odanacatib) in Prolonging Time to First Bone Metastasis in Men With Castration-Resistant Prostate Cancer, http://adisinsight.springer.com/trials/700034934.

^6^http://www.nndc.bnl.gov/.

^7^Carbobenzoxy-capped Phe-Lys(Cy5)-acyloxymethyl ketone. http://www.ncbi.nlm.nih.gov/pubmed/20641998.
